# Gender typicality of occupational aspirations among immigrant and native youth: the role of gender ideology, educational aspirations, and work values

**DOI:** 10.3389/fsoc.2023.1161131

**Published:** 2023-06-27

**Authors:** Ludovica Gambaro, Janna Wilhelm, Pia Sophia Schober

**Affiliations:** ^1^Department of Sociology, University of Tübingen, Tübingen, Germany; ^2^Federal Institute for Population Research, Wiesbaden, Germany

**Keywords:** gender segregation of occupations, intersectionality, occupational aspirations, work-related values, adolescence, immigrant background, Europe

## Abstract

The gender typicality of adolescents' occupational aspirations helps sustain occupational segregation, ultimately contributing to maintain gender stratification. According to sociological and psychological perspectives, adolescents develop occupational aspirations by drawing on their gender beliefs and work-related values. Yet few empirical studies have examined the contribution of these value orientations specifically to the gender typicality of occupational aspirations. Moreover, although children from immigrant backgrounds make up an ever-increasing share of school-age students, there is scant evidence on the gender typicality of their occupational aspirations relative to those of their majority peers. This study investigates variations in the gender typicality of occupational aspirations among adolescents from immigrant and non-immigrant backgrounds at around age 16. It also explores how the gender typicality of different groups' aspired occupations relates to differences in gender ideologies, in educational aspirations, and in the importance attributed to three work values: the possibility to earn high income, to help others, and to think and solve problems. Drawing on a harmonized survey from England, Germany, the Netherlands and Sweden, the analysis uses a sample of 8,574 adolescents, including 1,510 girls and 1,336 boys from immigrant backgrounds. Multinomial logistic regressions estimated the associations with aspired occupations, classified as masculine, integrated, feminine or ultrafeminine based on the proportion of women working in them. Results indicate that boys and girls of immigrant origin aspired to somewhat less gender-typical occupations than their majority peers. Among girls, these differences would be even larger if they were not suppressed by the more traditional gender ideologies held by girls from immigrant backgrounds. In terms of mediating mechanisms, our findings suggest that more ambitious educational aspirations may partly explain these differences. These findings indicate that distinguishing between multiple dimensions of adolescents' work-related values hint at different underlying mechanisms in the formation of adolescents' occupational aspirations.

## 1. Introduction

Significant gender differences in occupational aspirations persist: girls continue to favor female dominated occupations and boys are more likely to favor male dominated occupations (Correll, 2004; Sikora and Saha, 2009; Polavieja and Platt, 2014; Barrett, 2021; Stoet and Geary, 2022). Aspirations in adolescence inform education and vocational choices that are consequential for labor market outcomes (Barone, 2011; Mann and DiPrete, 2013). The gender typicality of early aspirations contributes to sustain the sorting of men and women into different occupations (Reskin, 1993; Charles and Grusky, 2004), which in turn explains a large share of the gender pay gap (Gerber and Cheung, 2008; Ochsenfeld, 2014; Levanon and Grusky, 2016; England et al., 2020). Within couples, women's lower wages relative to men further a more traditional division of paid and unpaid work (e.g., Schober, 2013; Grunow and Evertsson, 2016; Nitsche and Grunow, 2016), which ultimately depresses women's lifetime earnings and pensions (Sigle-Rushton and Waldfogel, 2007; Bettio et al., 2013). Gender-typical aspirations also reinforce traditional gender cultures in the workplace (Taylor, 2010) and, especially in men-dominated jobs, restrict choices of combining family care with careers.

This paper investigates adolescents' occupational aspirations, broadly defined as idealized goals expressing career interests and desires that are not necessarily limited by existing constraints. We focus specifically on the gender composition of the occupations adolescents aspire to, thus assessing to what extent adolescents' occupational interests mirror gender segregation in the workforce. Adolescence is an especially fruitful life stage to study, because boys and girls develop a more coherent worldview informed by their values and cultural orientations during this period (Kohlberg, 1969, 1976; Selman, 1980). Adolescents with more gender-typical preferences and aspirations have been found to choose gender-typical fields in post-secondary education (Morgan et al., 2013; van der Vleuten et al., 2016) and to work in gender-typical occupations as adults (Okamoto and England, 1999; Polavieja and Platt, 2014). Life-course scholars have shown how value orientations, particularly those explicitly related to work, influence occupational aspirations during adolescence, and actual occupational attainment in adulthood (Schoon, 2001; Schoon and Parsons, 2002; Johnson and Mortimer, 2011; Schoon and Eccles, 2014). Indications that value orientations underpin occupational aspirations and choices also come from the stratification theory of gender essentialism. Charles and Bradley (2009) and Cech (2013) observed how enduring beliefs about “inherent” differences between men and women and cultural endorsement of self-expression contribute to young's people education and occupational choices, thus sustaining gender segregation in post-industrial societies. Building on these insights, this paper examines how a range of value orientations pertaining to work and gender are associated with adolescents' occupational aspirations.

As Western societies become more diverse and as children from immigrant backgrounds make up an ever-increasing share of students (OECD, 2018), it is essential to also consider the interplay between gender and ethnicity or immigrant background. Adolescents from immigrant backgrounds are likely to be exposed to conflicting sets of values, especially if their parents have been socialized in a different cultural context (Idema and Phalet, 2007; de Valk, 2008; Kogan, 2018), with potential repercussions on their early occupational aspirations. For example, there is evidence that adolescents from several immigrant groups in European countries hold more traditional gender beliefs compared to their majority peers (Sánchez Guerrero and Schober, 2021). Children of immigrants have also consistently been found to attribute greater importance to educational achievement, something that translates into higher educational ambitions (Nauck and Lotter, 2015; Dollmann, 2017; Plenty and Jonsson, 2021). By potentially increasing values diversity, the presence of children of immigrants also offers the chance to understand whether and how values influence the gender typicality of occupational choices.

The present study extends the literature by systematically examining variations in the gender typicality of occupational aspirations among adolescents from immigrant and non-immigrant backgrounds in four European countries. Drawing on a representative sample of students from different backgrounds in England, Germany, the Netherlands, and Sweden, we explore how their evaluative orientations in different domains relate to the gender typicality of their aspired occupations. Specifically, we investigate beliefs about the appropriate gender division of paid and unpaid work, educational aspirations, and the importance attributed to a job affording high income, the possibility to help others, or the possibility to think and solve problems. Despite the theoretical importance attributed to gender beliefs (Charles and Bradley, 2009) and work-related values as “influencing the attractiveness of different goal objects and, consequently, the ability to attain these goals” (Eccles and Wigfield, 2002), few studies have examined the role of gender ideologies and work-related values in relation to the gender typicality of occupational aspirations and choices (Marini et al., 1996). By investigating value orientations across different domains, we also address, albeit incompletely, the inherent multidimensionality of values and beliefs, offering insights into their potentially different relevance for occupational aspirations. By using data from four different countries, we are also able to assess whether these patterns of associations unfold similarly across four countries, thus providing indirect evidence for the potential role of different macro-contexts. Although our study is exploratory, we contribute to the literature by offering a comprehensive account of the intersectional patterns of gender-typical occupational aspirations as well as the role of gender beliefs, educational aspirations, and different work values in four different countries, thereby improving our understanding of occupational gender segregation.

## 2. Conceptual framework and existing evidence

### 2.1. The formation of adolescents' occupational aspirations

To understand the social psychological processes that may facilitate gender occupational segregation, it is helpful to draw on psychological theories (Bussey and Bandura, 1999; Eccles and Wigfield, 2002), which generally posit that occupational choices reflect people's effort to implement their preferred self-concepts. Gottfredson's theory of circumscription and compromise falls into this mold and seems particularly relevant here, as it concentrates on adolescents' sense of social self and on gender in particular, providing helpful insights into how gender differentiation in aspirations occurs.

According to Gottfredson (1981), three sequential processes underpin occupational choices: (i) the development of self-concepts and of occupational images; (ii) the identification of desirable options, based on their compatibility with one's self-concept, in which occupational choices are narrowed down or circumscribed; (iii) the formation of actual aspirations, which starts in the early teenage years, with focus shifting to societal valuations and the prestige of different occupations and considering one's internal characteristics such as motivation, values, and ability. Gender, which is incorporated in the self-concept, influences which occupational choices are deemed compatible. In making compromises, adolescents are argued to prioritize congruence with their gender self-image above correspondence with interests or ambitions, because, according to Gottfredson (1981, p. 572), “gender is the most strongly protected aspect of self”.

While Gottfredson's framework offers a relevant description of occupational aspirations' development, we draw on a more sociological notion of gender and on specific theoretical insights to spell out how gender may influence occupations' desirability. We also extend Gottfredson's framework by considering the interplay between gender and immigration status. We elaborate on these aspects in the next two subsections.

### 2.2. Gender and occupational aspirations

Based on a sociological understanding of gender as a social structure, we expect gender to permeate the formation of occupational aspirations, reflecting material and cultural factors at the individual, interactional, and institutional level (Risman, 2004, 2017). Gender is therefore not only a major element of the self-concept, but also an integral part of adolescents' views of paid work—how it ought to be divided between the genders and its affordances. We look at these two aspects by distinguishing between gender ideologies and work-related values and discussing each of them in turn.

Following Davis and Greenstein (2009), we define gender ideologies as attitudes about the division of paid work and family responsibilities based on the notion of separate gendered spheres. Individuals are considered to be more traditional if they tend to support such separation and more egalitarian if they favor an equal division. Although adolescents have not yet engaged in any division of work, they have been exposed to the arrangements practiced by their parents, with potential repercussions on their occupational aspirations. Studies of the intergenerational transmission of gendered occupational aspirations give some support to this idea. Examining a sample of British adolescents surveyed between 1994 and 2008, Polavieja and Platt (2014) identified significant associations between fathers' involvement in domestic work and boys aspiring to less male-dominated occupations. Equally, Busch-Heizmann (2014) reported that in Germany, male adolescents whose parents had a more traditional division of labor aspired to more male-dominated occupations. Drawing on the same longitudinal data from Germany, Law and Schober (2021) also found evidence related to girls: they were more likely to aspire to female-dominated occupations if their mothers had never been in employment since their birth.

Studies directly investigating the role of adolescents' gender ideologies in the second decade of the 2000s confirmed these patterns. Using the same data as we do, van der Vleuten et al. (2016) found that, in the Netherlands, male adolescents holding more traditional gender ideologies were less likely to choose stereotypically feminine subjects, ultimately choosing more male-dominated educational tracks. A similar finding emerged from data on adolescents in Germany: male students who held more traditional gender ideologies were found to be less likely to aspire to female-dominated jobs relative to male-dominated jobs (Chesters, 2021). Conversely, female students with more traditional beliefs were less likely to aspire to a gender-neutral or male-dominated job relative to a female-dominated one (Chesters, 2022). Lawson et al. (2018), in their longitudinal study of gendered vocational development in the US, confirmed that adolescents who expressed more traditional gender ideologies were more likely to make more gender-typical occupational choices.

Although these findings are not surprising, the underlying mechanisms are not always spelled out. The theory of compensating differentials applied to gender segregation suggests that women seek jobs that offer greater flexibility to reconcile paid work with care responsibilities, ultimately resulting in relatively lower pay (Glass and Fujimoto, 1995). Although different studies have questioned the notion that female-dominated jobs are invariably more family-friendly than male-dominated ones (Glauber, 2011; Chung, 2019; Magnusson, 2021), it could be that girls perceive occupations disproportionally employing women as more suitable to accommodating care duties within the family.

An alternative explanation is instead offered by the stratification theory of gender essentialism, which argues that post-industrial labor markets offer a highly diversified range of occupations and abundant female-typed service jobs (Charles and Bradley, 2009; Cech, 2013). This, in turn, gives adolescents with traditional gender ideologies ample opportunity to enact traditional roles (Charles and Bradley, 2009), even without explicitly or consciously drawing on such beliefs (Cech, 2013). More specifically, adolescents are argued to consider and frame their career decisions as free expressions of their individuality. In a context in which essentialist beliefs are widespread and in which self-expression and self-realization are highly valued, self-expression will reinforce gender-typical choices in terms of curricula, fields of study and occupations (Charles and Bradley, 2009; Cech, 2013; Charles, 2017). Under which circumstances does self-expression trump more instrumental concerns, such as money and security? While cross-country evidence supports the idea that higher affluence is associated with more strongly gendered self-expression, evidence on the role of individual circumstances is lacking.

Adolescents' views of paid work will also be influenced by their work-related values, here understood as beliefs about desirable goals and behaviors in the work setting (Ros et al., 1999; Cemalcilar et al., 2018; Kraaykamp et al., 2019). While gender ideologies may be associated with the centrality given to paid work relative to family responsibilities, work-related values are likely to orientate adolescents in relation to the type of occupation chosen. Substantial longitudinal research on young adults has indeed indicated that work-related values play a vital role in occupational outcomes in adulthood (Johnson and Mortimer, 2011), and yet their potential relation to occupational gender segregation has been little studied (Johnson, 2001).

We assume that the endorsement of different values will be influenced by gender schemas. Following Charles and Bradley (2009) and Cech (2013), we expect some gender patterning of work-related values, as they allow adolescents to express their gendered selves and their potentially essentialist beliefs about men's and women's predispositions for different roles. In particular, we focus on three work-related values that appear relevant for the gender typicality of occupational choice: “valuing income”, “valuing helping others”, and “valuing thinking and solving problems”. These values are helpful because they are not overly specific and correspond well with gender-stereotypical expectations in society. Money and breadwinning are associated with masculinity; nurturing, helping others and maintaining positive relationships are associated with femininity (Weisgram et al., 2011). Thinking and solving problems overlaps with being analytical and mathematical, which is stereotypically a male trait (Correll, 2001; Ridgeway and Correll, 2004; Levanon and Grusky, 2016).

Empirical findings on how these work values relate to occupational aspirations are mixed, with more consistent results in relation to pro-social values and no evidence in relation to thinking and solving problems. While men and women in the past tended to endorse different work-related values, gender differences have become less obvious in younger cohorts and/or more recent periods (Johnson, 2001; Gallie, 2019), in line with historical changes in female employment rates. Among those studying adolescents specifically, Marini et al. (1996) examined gender differences in job values among high school seniors (17–18-year-olds) in the US from 1976 to 1991. Young women were found to attach relatively more importance to altruistic rewards than men, and to “being helpful to others” in particular. However, among younger cohorts, there was no gender gap in the value placed on pay, advancement opportunities, and prestige. More recent experimental work by developmental psychologists has confirmed the higher endorsement of altruistic values among teenage girls relative to boys, but has also shown that valuing altruism was associated with female-dominated occupations among girls but not among boys (Weisgram et al., 2010). The differential effect was attributed to a gendered understanding of “helping others”: as exercising authority among boys and as exercising feminine role traits (being compassionate and sensitive) among girls (Pryor, 1983; Weisgram et al., 2011). Among 6- to 11-year-old children, boys were found to prioritize jobs that afforded money significantly more than those affording other values and ranked jobs that afforded money significantly higher than did girls (Hayes et al., 2018).

Based on these insights and findings, we contend that the gender typicality of boys' and girls' occupational aspirations will be affected by adolescents' orientations concerning the gendered division of labor and their desired rewards from work. In line with sociological theory, we extend Gottfredson (1981) concept of the gendered self, which refers to the prescribed gender role an individual inhabits, to look at gendered norms about relationships: between men and women in the household—gender ideology—and between the individual and society at large—work values.

### 2.3. The intersection of gender and immigrant background

In the present study, we also consider how gender may interact with social origin and consider immigrant background in particular. The interplay between immigrant background and gender is a fruitful angle of analysis, both empirically and conceptually. Empirically, it better accounts for the diversity in Western societies, especially among adolescents. We are aware of only one study charting how gender and migration intersect in explaining occupational aspirations in Germany, pointing to a less pronounced gender typicality in the aspirations of students from immigrant backgrounds, especially girls, compared to their majority peers (Wicht and Siembab, 2022). The study found some support for the notion that immigrant youth are influenced by multiple cultural contexts and that countries of origin can also serve as a frame of reference. However, the authors did not directly explore the potential role of educational aspirations or value orientations.

Conceptually, it is fruitful to examine the interplay between immigrant background and gender because a large body of migration research has documented differences in attitudes and value orientations between majority and minority groups of the first or second generation (e.g., Logan and Shin, 2012; Röder, 2014; Röder and Mühlau, 2014; Nauck, 2023). This, in turn, raises the question of whether potential differences in gender ideologies and work-related values result in distinct gender dynamics in occupational aspirations across groups of immigrant and non-immigrant origin.

A large proportion of adult immigrants to Western Europe have been socialized in less gender-egalitarian countries and tend to hold more traditional values than the average person in the country of arrival (Kogan, 2018). Additionally, some immigrant groups exhibit larger gender differences in labor market participation than the majority population, resulting in a more traditional division of labor at home (Diehl et al., 2009; Schieckoff and Diehl, 2021). Acculturation processes tend to reduce these differences (Röder and Mühlau, 2014), although not necessarily uniformly across genders. Young women appear to be more responsive to the gender egalitarianism of receiving countries than their male peers, possibly because of their larger potential gains from it (de Valk, 2008). Investigating adolescents from the same four European countries examined here, Sánchez Guerrero and Schober (2021) reported that within each gender group, adolescents from the majority population held more egalitarian beliefs than their minority counterparts.

Although work orientation is assumed to be an important driver of migration (for a discussion, see Polavieja et al., 2018), there is no evidence on differences between immigrant and native youth on the endorsement of certain work values relative to others. There is, however, some evidence on differences by socio-economic background. Johnson and Mortimer (2011), for example, show that whereas fulfilling jobs are universally valued across social classes, young people from more advantaged families hold weaker extrinsic orientations than their less privileged peers. Because immigrant status in Europe often overlaps with lower socio-economic status, it could be that immigrant adolescents place greater importance on values such as pay or advancement opportunities than their more advantaged peers.

In examining differences in value orientations between adolescents of migrant and non-migrant origin, we need to account for orientations toward education. Specifically, we need to address the well-documented empirical finding that children of immigrants express higher educational aspirations and make more ambitious choices than their peers who perform equally at school and whose parents have a similar socioeconomic background (van De Werfhorst and Van Tubergen, 2007; Kristen and Dollmann, 2009; Jackson et al., 2012; Nauck and Schnoor, 2015; Hadjar and Scharf, 2019; Dollmann and Weißmann, 2020). While educational aspirations have straightforward, and indeed proven, implications on the status of the aspired occupations, their effect of the gender-typicality of aspired occupations is less obvious (Prix and Kilpi-Jakonen, 2022). Gender typicality and hierarchical ordering of occupations partly overlap: more integrated occupations generally tend to have higher prestige and/or pay, especially relatively to female-dominated ones, even when qualification requirements are similar (Estévez-Abe, 2006; England et al., 2007; Magnusson, 2009, 2013; Grönlund and Magnusson, 2013; García-Mainar et al., 2018).

Thus, valuing monetary rewards and having high educational aspirations may result in less gender-typical choices among immigrants relative to natives. Likewise, more instrumental concerns, such as money and security, may leave less space for self-expression, limiting the influence of preferences for a more traditional division of roles among immigrants. Evidence from the UK based on cohorts born in the 1990s and early 2000s points in this direction, with minority boys and girls more likely to aspire to well-paid jobs relative to majority children of the same sex (Platt and Parsons, 2017). We would therefore expect adolescents from immigrant backgrounds to hold less gender-typical aspirations, albeit possibly with some asymmetries between boys and girls.

### 2.4. The present study and hypotheses

The aim of this article is to investigate the degree of gender typicality in the occupational aspirations of girls and boys from immigrant and non-immigrant backgrounds. Following Gottfredson (1981), occupational aspirations are understood as the ideally preferred occupation expressed at any one point in time. In choosing to investigate 15-year-old adolescents, we focus on a time when aspirations reflect young people's self-concept, including interests and perceived place in society, but also more concrete knowledge of different occupations and institutional restrictions to achieving their occupational goals.

**Figure 1:**
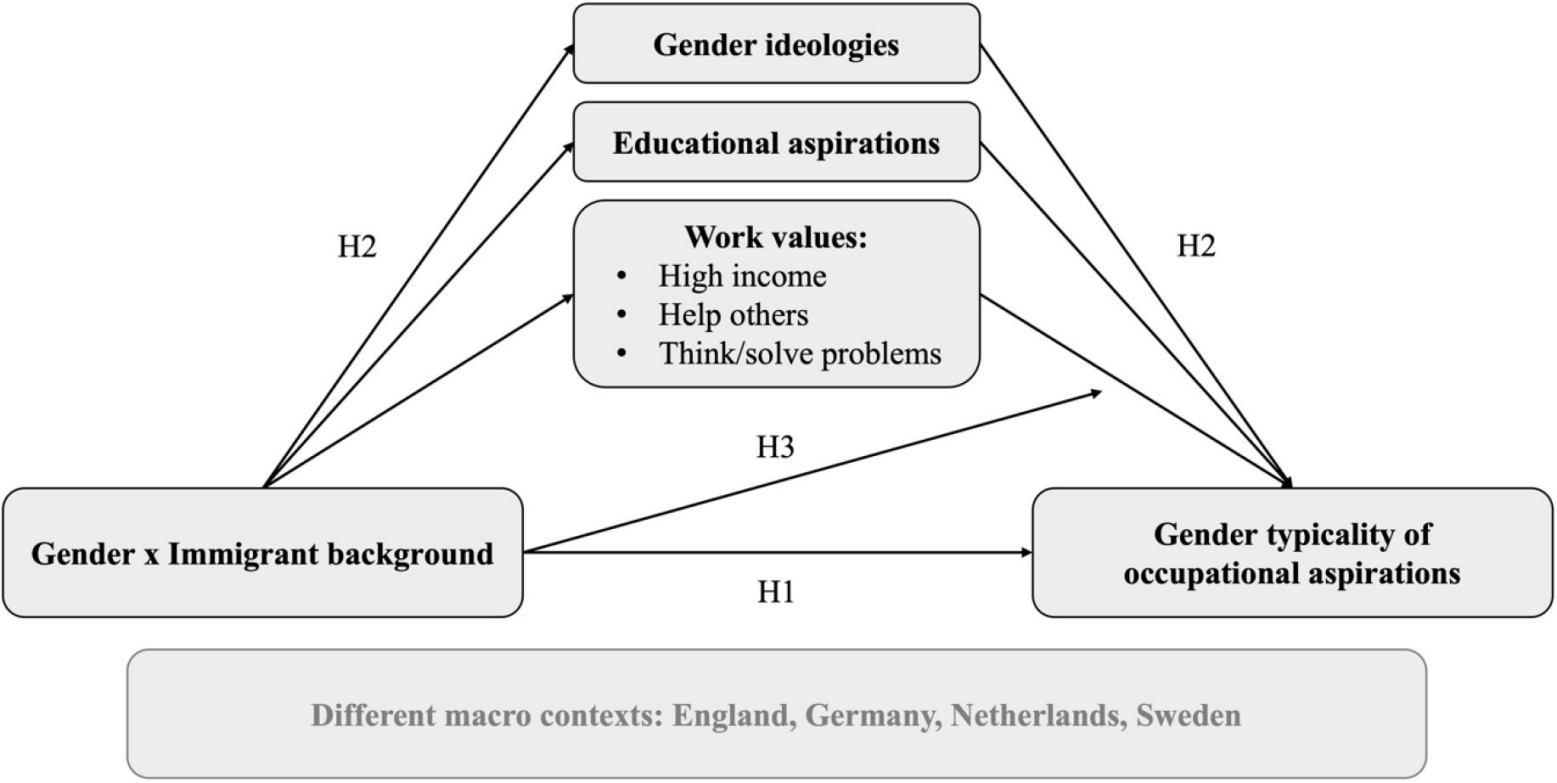
Conceptual model and hypotheses.

Our interest lies in the gender typicality of aspirations, and thus the extent to which girls express a preference for female-dominated occupations and boys for male-dominated ones. We also pay attention to mechanisms that do not work symmetrically and may reinforce or reduce aspirations specifically for female-dominated or male-dominated occupations, and which may be more or less gender-typical depending on the identity of the respondent. In exploring occupational aspirations, we assess the predictive power of different values and beliefs: gender ideologies, educational aspirations, and three specific work-related values that correspond well with gender stereotypes: valuing income, valuing helping others, and valuing thinking and solving problems (which hereafter we collectively refer to as “work values”). To guide the analysis, we propose the following research questions and hypotheses and refer to the diagram in Figure 1.

First, we aim to explore how gender and immigrant background map onto the gender typicality of adolescents' occupational aspirations. While we expect girls (boys) to aspire to female-dominated (male-dominated) occupations (Gottfredson, 1981; Risman, 2004, 2017), we are interested in understanding whether there are variations in the gender typicality of occupational aspirations associated with adolescents' immigrant background within the two gender groups. As limited empirical evidence suggests that immigrant-origin youth, particularly girls, are more likely to orient themselves away from gender-typical occupations (Platt and Parsons, 2017; Wicht and Siembab, 2022), we expect the following:

*Hypothesis 1a: Immigrant-origin adolescents hold less gender-typical occupational aspirations than their non-immigrant peers*.

*Hypothesis 1b: The differences between adolescents from non-immigrant vs. immigrant backgrounds are larger among girls*.

Second, we aim to examine the influence of gender ideologies, educational aspirations, and the three work values on the gender typicality of occupational aspirations—initially, without taking into account gender and immigrant background. Are these values and beliefs associated overall with higher or lower levels of gender typicality in aspirations? As set out above, more traditional gender ideologies are likely to positively correlate with the gender typicality of occupational aspirations, for instance, because female-dominated jobs are frequently conceived as being easier to combine with family responsibilities (Chung, 2019) and because they facilitate the expression of the gendered self (Charles and Bradley, 2009; Cech, 2013). Furthermore, female-dominated occupations include more interactive and nurturing tasks (Grunow and Veltkamp, 2016). As a result, young people who attach greater value to helping others in their job are more likely to aspire to female-dominated occupations. Aspirations toward tertiary educational qualifications will facilitate access to high-status professional occupations, which tend to be more integrated than male-dominated occupations in the industrial sector or female-dominated in lower status services. Therefore, young people who aspire to higher educational qualifications are less likely to aspire to gender-typical occupations. Pay levels tend to be lower in female-dominated occupations, often also when comparisons are made holding the level of educational qualifications constant (e.g., Magnusson, 2009; Leuze and Strauß, 2016; Krueger et al., 2022). Valuing income and thinking/solving problems have also been found to be more strongly associated with masculinity (Weisgram et al., 2011). We therefore formulate the following hypotheses:

*Hypothesis 2a: Adolescents with more gender-egalitarian ideologies are more likely to aspire to integrated compared to gender typical occupations*.

*Hypothesis 2b: Adolescents with higher educational aspirations are more likely to aspire to integrated compared to gender typical occupations*.

*Hypothesis 2c: Adolescents who value high income and thinking/solving problems more are less likely to aspire to female-dominated compared to other occupations*.

*Hypothesis 2d: Adolescents who value helping others more are more likely to aspire to female-dominated compared to other occupations*.

Third, we hypothesize that educational aspirations and valuing a high income partly mediate the differences between young people from immigrant and non-immigrant backgrounds within both gender groups, whereas gender ideologies are likely to act as suppressors of these differences. Immigrant-origin youth have been found to report higher educational aspirations and are likely to attach greater value to high income, as they are more likely to come from less privileged backgrounds and thus have a greater need to focus on pay and career opportunities (Johnson and Mortimer, 2011). Accounting for these two sets of values is likely to reduce differences in the gender typicality of occupational aspirations between youth of immigrant and non-immigrant origin. By contrast, the more traditional gender ideologies held on average by youth of immigrant origin are likely to contribute to their aspiring to more gender-typical occupations than youth from non-immigrant backgrounds and may result in a suppressor effect. Due to a lack of previous empirical evidence on variations in valuing helping others and thinking/solving problems by immigrant background, we refrain from formulating any hypotheses regarding possible mediation relationships with these work values.

*Hypothesis 3a: Higher educational aspirations and strongly valuing income partly explain the less gender-typical occupational aspirations among immigrant-origin adolescents compared to their non-immigrant peers*.

*Hypothesis 3b: More traditional gender ideologies suppress the difference in the gender typicality of occupational aspirations between immigrant-origin and non-immigrant origin youth*.

Finally, we ask whether the associations between gender ideology, educational aspirations, and work values on the one hand and the gender typicality of occupational aspirations on the other vary in strength across immigrant and gender groups.

Compared to previous work on the gender typicality of adolescents' occupational aspirations, our approach has three main innovative features. First, it looks at gender and immigrant status together, allowing for potentially differential gender dynamics across groups. Second, it investigates how multiple dimensions of adolescents' work-related values relate to occupational aspirations. The distinction between educational aspirations, work values, and gender ideologies is instructive, as it can potentially hint at different underlying mechanisms. Educational aspirations partly capture the vertical dimension of gender segregation, connected to the gender typicality of occupations (England, 2010; Platt and Parsons, 2017), whereas intrinsic work values partly overlap with horizontal gender segregation (Levanon and Grusky, 2016). The third innovative feature of our study is that it includes data from four different countries, which allows for exploring to what extent differences by gender and immigrant background with respect to occupational aspirations and potential influences are similar or vary across countries.

## 3. Institutional contexts

Occupational choice processes cannot be described simply in terms of individual differences in orientations and/or constrains, but need to be conceptualized as embedded in overlapping contexts, including more distal macro-contexts (Schoon and Parsons, 2002). We know from large cross-national comparisons that the strength of gender differentiation tends to be larger in richer nations (Stoet and Geary, 2022), suggesting that differences in underlying contextual factors have a bearing on the gender typicality of adolescents' aspirations.

In this study, we maintain a focus on individual-level factors, but seize the opportunity offered by harmonized data from England, Germany, the Netherlands, and Sweden to explore potential variations in the patterns of associations. We refrain from formulating specific predictions on cross-national variations in how gender and immigrant background relate to occupational aspirations and on the role of gender ideologies, work values and educational aspirations. Instead, we briefly highlight some salient differences across the four countries, which can be systematically explored in future research.

At a broad level, the four countries are all rich, postindustrial welfare states, but display variations in their policy support for a gender-equal division of responsibilities among parents, in the structure of their labor market and educational systems, and in their immigration histories. Sweden's strong culture of gender equality has long been institutionalized in the extensive provision of support services for working mothers and caring fathers (Goldscheider et al., 2011). Differently, England and the Netherlands have implemented equal pay and antidiscrimination laws to provide women and men with equal access to the labor market, without actively supporting gender equality in the private sphere (Chang, 2000; Grunow and Veltkamp, 2016). This policy orientation, combined with a strong culture promoting autonomous individual choices, is likely to value the expression of gender-typed self-conceptions. At the other end of the spectrum, public policies in West Germany have traditionally encouraged women to structure their employment around family obligations (e.g., by taking long maternity leave periods after childbirth) and provided little childcare support, reflecting strong familialist norms. Despite a paradigm shift since the mid-2000s (Stahl and Schober, 2018), adolescents born at the turn of the millennium had been mainly exposed to Germany's traditional family-centered policies. As a result, adolescents' gender ideologies have been found to be most egalitarian in Sweden and least so in Germany (Sánchez Guerrero and Schober, 2021). Conversely, work values reflecting individual self-expression are more likely to be acted upon in England and the Netherlands, resulting in contrasting influences on the gender typicality of occupational aspirations.

Besides being immersed in distinct gender cultures, the adolescents sampled in the four countries were confronted with labor markets characterized by different earnings distributions and occupational structures (Eurofound, 2017). England stands out for its earnings differential, with a substantial tertiary education wage premium (Strauss and Maisonneuve, 2009) and a larger prevalence of low-wage employment (Lloyd et al., 2008; Gautié and Schmitt, 2010), especially in the female-dominated service sector (Grimshaw and Rubery, 2007). At the opposite end is Sweden, where wage dispersion is small and a more extensive welfare system mitigates the importance of market earnings for meeting families' needs (Eurofound, 2017). Additionally, studies of care occupations—which invariably display high levels of feminization—indicate that in Sweden, care sector workers and women in particular enjoy a large wage premium (Budig and Misra, 2010; Addati et al., 2018). Germany and the Netherlands are in between, although over the past two decades, the share of low-wage workers has also increased in these countries (OECD, 2011, 2023).

The four countries' education systems differ in their level of selectivity and stratification. In Germany and the Netherlands, students are sorted into hierarchically ordered tracks with very different curricula at age 10 and 12, respectively, whereas Sweden and England have comprehensive school systems (Nauck, 2023). Vocational education is more important in Sweden, Germany and the Netherlands than in the UK, effectively providing vocational graduates with well-paid and well-regarded jobs. We expect therefore that occupational aspirations may be more realistic in Germany and the Netherlands, where students have already been allocated to different educational tracks—we take this point into account in the empirical analysis. At the same time, the gender typicality of aspirations may be stronger where vocational education is more important: vocational education and training systems are more closely linked to occupational identities than general academic studies, and are often more established in manufacturing and commercial sectors that have been historically male-dominated, as in the German case (Haasler and Gottschall, 2015).

The four countries also vary in their immigration and immigrant incorporation policies (Koopmans, 2013; Drouhot and Nee, 2019). Several studies have argued that legal or economic disadvantages experienced by immigrant groups are likely to slow down acculturation, which offers smaller economic returns in such cases (Drouhot and Nee, 2019). Sweden, England, and the Netherlands developed antidiscrimination and multicultural policies during immigration waves between the 1950s and 1980s. Naturalization policies varied and were most generous in England and the Netherlands for immigrants from former colonies. Germany viewed most of its immigrant population as temporary guest workers, pursued very restrictive citizenship and multicultural policies, and introduced antidiscrimination policies only in 2006 (Joppke, 2007; Koopmans, 2013; Drouhot and Nee, 2019). Germany's restrictive policies (Koopmans, 2013) may preserve differences in gender and work values and occupational aspirations among most groups of immigrants more than the multicultural policies in the other countries.

Overall, a multitude of cultural, economic, and institutional characteristics can differentially affect adolescents' occupational aspirations in the four countries. However, the small number of countries does not allow for identifying the specific role of different institutional characteristics (for a similar argument, see Nauck, 2023). Nevertheless, we take advantage of the increased variability at the contextual level to also explore whether the patterns of associations vary across countries.

## 4. Data, variables, and method

### 4.1. Data and analytical sample

Data for this study were drawn from the Children of Immigrants Longitudinal Survey in Four European Countries (CILS4EU) that followed students from Germany, England, the Netherlands, and Sweden starting in 2010/11, when they were 15 years old (Kalter et al., 2016a,b). From national lists of eligible schools, 480 schools were selected with probabilities proportional to the size of the school. Schools with large immigrant proportions were oversampled to include enough students from immigrant backgrounds. After adding replacement schools that were similar to non-responding schools, the response rate of schools differed rather strongly across countries: 65% in England, 77% in Sweden, 92% in the Netherlands, and 99% in Germany. Within the sampled schools, two classes were selected at random from the country-specific grades covering the target age group. All students within those classes were asked to participate (*n* = 18,716). Class response rates were almost 100% in all countries and student response rates were between 80% in England and 91% in the Netherlands (CILS4E, 2016).

We restricted our analysis to Wave 2, when information on the main variable of interest—aspired occupation—was elicited. Wave 2 was conducted in 2011/12, when respondents were 16 years old on average. Those students who were no longer in school (around 3% of the sample) were dropped, as their occupational aspirations might have already translated into actual apprenticeship or employment choices. We also excluded from the analytical sample those students who did not report any aspired occupation-−31% of the sample, *n* = 9,603. Whereas a previous study on adolescents in England indicated that uncertainty in career aspirations was associated with lower socio-economic background and lower prior school achievement (Gutman et al., 2012), additional analyses conducted on our sample showed that having more highly educated parents and higher educational aspirations, holding more egalitarian gender ideologies, and valuing a high income were significantly related to a higher probability of non-response to the occupational aspiration question. Gender was not related to non-response in the overall sample, but boys were more likely to not respond than girls in England. Also, respondents from England and the Netherlands had significantly more missing values compared to Germany, which had the lowest non-response level on this question. Our results thus do not fully reflect the dynamics of occupational aspirations among adolescents with relatively highly educated parents, especially in England, where boys may be underrepresented, and the Netherlands.

The level of missing values on the covariates was low overall, with around 3% missing values for gender ideologies and for the work values items, and 8% for educational aspirations. We thus implemented listwise deletion. We ended up with a non-weighted sample of 8,574 respondents, including 1,803 from England, 2,330 from Germany, 1,924 from the Netherlands, and 2,517 from Sweden. Before applying weights to correct for the oversampling of immigrant-origin adolescents, 2,846 respondents (33% of the overall sample) indicated having an immigrant background.

### 4.2. Measures and descriptive statistics

#### 4.2.1. Dependent variable

CILS4EU asked adolescents to name the one “occupation they would like to have as an adult”. The question captured idealistic aspirations, as opposed to evaluations of which occupation they were likely to hold as adults. Nonetheless, and following Gottfredson's account, even idealistic aspirations reflect, albeit implicitly, respondents' understanding of different occupations, including opportunities and restrictions to achieve them.

To create a measure of *gender typicality* of occupational aspirations, we referred to the gender of workers who were typically employed in that occupation. An alternative way would be to base the definition on the characteristics of the occupation itself; for example, the jobs of mathematicians, actuaries and statisticians would be classified as masculine because they entail the use of mathematics, which has a strong masculine attribution. But, as discussed and experimentally investigated by Weisgram et al. (2010), occupational gender segregation of jobs shapes perceptions of the traits required for occupational roles, so that it is difficult to empirically distinguish between the two. In choosing a definition based on the proportion of women and men in each occupation, we consider the overrepresentation of one gender in certain occupations to reinforce adolescents' ideas about what is typically feminine and masculine, ultimately influencing their aspirations.

Operationally, the CILS4EU data team coded the answers by respondents according to the International Standard Classification of Occupations (ISCO) 2008 (ILO, 2012). We derived information on the shares of women in all occupations from national statistical offices (Office for National Statistics, 2011; Official Statistics of Sweden, 2014; Statistisches Bundesamt, 2014) and converted to ISCO 2008 if a national coding system had been used. For the Netherlands, we used information from the European Labor Force Survey (Eurostat, 2020). The share of women in an occupation mostly referred to 2011, with the exception of Sweden, where the data was from 2014 instead. As we used the reduced off-site version of CILS4EU that only reveals 2-digit sub-major groups instead of the more detailed 4-digit ISCO units, we asked the survey's data services team to run a code file provided by us, merge the data on female share with the 4-digit responses on-site, and create a categorical gender share variable, which they sent back to us. The categorical variable differentiated among masculine (< 25% women), integrated (25 to 49.9% women), feminine (50 to 74.9% women), and ultra-feminine occupational aspirations (≥75% women). We followed Hakim (1993) and Moulton et al. (2018) in labeling occupations asymmetrically to highlight occupations with a female majority; this practice also reflects findings from experimental psychology indicating that pre-adolescents tend to rate jobs as gender neutral that are in fact male-dominated (Liben et al., 2001; Teig and Susskind, 2008). The occupations were classified similarly across countries, with only minor differences. The gender-balanced category covers occupations such as specialist medical doctors, lawyers, or university instructors, while the masculine category included different kinds of engineering professionals or software developers. Only in Sweden were some of the engineering professions categorized as gender-balanced or even feminine. Occupations categorized as feminine in all countries included secondary school teachers, psychologists, and social workers, whereas the ultra-feminine category always comprised care-focused occupations such as nurses and midwives as well as primary school and early childhood teachers.

As shown in Table 1, 30% of the weighted full sample aspired to masculine, 27% to integrated, 28% to feminine, and 15% to ultra-feminine occupations. Descriptive analyses showed relatively similar distributions across countries (not shown). Across subgroups, girls aspired to feminine and ultra-feminine occupations more often than boys, with non-immigrant girls aspiring significantly more often to ultra-feminine occupations and significantly less often to integrated and feminine jobs compared to immigrant-origin girls. Boys, on the other hand, aspired to masculine jobs a lot more often than girls. Among boys, those from non-immigrant backgrounds aspired to masculine jobs significantly more often than those from immigrant backgrounds (Table 1).

**Table 1:** Descriptive statistics by subgroup (weighted sample).

	**Mean (SD)**					**Difference**
		**Boys**		**Girls**		
	**Full sample**	**NB**	**IB**	**NB**	**IB**	
Pooled sample	1.00 (0.00)	0.40 (0.49)	0.08 (0.27)	0.42 (0.49)	0.09 (0.29)	-
England	0.22 (0.41)	0.19 (0.39)	0.25 (0.43)	0.22 ( 0.41)	0.27 (0.44)	-
Germany (non-academic track)	0.13 (0.34)	0.13 (0.33)	0.20 (0.40)	0.12 (0.32)	0.16 (0.37)	-
Germany (academic track)	0.14 (0.34)	0.14 (0.35)	0.10 (0.29)	0.15 (0.35)	0.12 (0.32)	-
Netherlands (non-academic track)	0.18 (0.39)	0.22 (0.42)	0.13 (0.33)	0.17 (0.38)	0.09 (0.28)	-
Netherlands (academic track)	0.08 (0.27)	0.07 (0.26)	0.03 (0.17)	0.10 (0.30)	0.06 (0.23)	-
Sweden	0.25 (0.43)	0.24 (0.43)	0.30 (0.46)	0.24 (0.43)	0.31 (0.46)	-
OA: Masculine	0.30 (0.46)	0.55 (0.50)	0.45 (0.50)	0.09 (0.29)	0.07 (0.25)	^a^
OA: Integrated	0.27 (0.44)	0.24 (0.43)	0.30 (0.46)	0.27 (0.44)	0.33 (0.47)	^b^
OA: Feminine	0.28 (0.45)	0.19 (0.39)	0.22 (0.41)	0.36 (0.48)	0.42 (0.49)	^b^
OA: Ultra-feminine	0.15 (0.35)	0.02 (0.15)	0.03 (0.18)	0.28 (0.45)	0.18 (0.38)	^b^
Gender ideologies (0–1)	0.75 (0.38)	0.65 (0.41)	0.61 (0.38)	0.86 (0.32)	0.79 (0.34)	^b^
EA: < Upper sec. school degree	0.11 (0.31)	0.13 (0.34)	0.10 (0.30)	0.10 (0.30)	0.06 (0.24)	^b^
EA: Upper sec. school degree	0.25 (0.43)	0.31 (0.46)	0.19 (0.39)	0.22 (0.41)	0.15 (0.35)	^a, b^
EA: University degree	0.65 (0.64)	0.55 (0.50)	0.71 (0.45)	0.68 (0.47)	0.79 (0.40)	^a, b^
WV: High income (1–4)	3.16 (0.60)	3.20 (0.59)	3.43 (0.60)	3.05 (0.59)	3.28 (0.62)	^a, b^
WV: Help others (1–4)	3.08 (0.74)	2.88 (0.72)	3.04 (0.78)	3.22 (0.70)	3.40 (0.68)	^a, b^
WV: Think/solve problems (1–4)	3.13 (0.68)	3.12 (0.70)	3.22 (0.71)	3.08 (0.65)	3.30 (0.69)	^a, b^
Parents without tertiary education	0.60 (0.49)	0.62 (0.49)	0.65 (0.48)	0.59 (0.49)	0.56 (0.50)	ns
Parents with tertiary education	0.40 (0.49)	0.38 (0.49)	0.35 (0.48)	0.41 (0.49)	0.44 (0.50)	ns
*Non-weighted N*	8,574	2,743	1,336	2,985	1,510	-

NB, non-immigrant background; IB, immigrant background; OA, occupational aspirations; EA, educational aspirations; WV, work values. Last column reports whether the difference between non-immigrant and immigrant-origin boys (a) and girls (b) is statistically significant (*p* < 0.05 level); or not significant (ns).

#### 4.2.2. Independent variables

To examine the intersection of gender and migration status, we created a variable with four categories: girls and boys from immigrant and non-immigrant backgrounds. In what follows, we sometimes refer to them collectively as *subgroups*. As previous research points to great similarity of immigrant-origin and non-immigrant individuals in many social values and practices by the third generation (Heinrich-Böll-Stiftung, 2010; Logan and Shin, 2012), we categorized those up until the 2.75^th^ generation as adolescents from immigrant backgrounds, which includes all respondents with at least one parent and three grandparents born abroad. In the final sample, the (weighted) share of adolescents of immigrant descent ranges from about 13 percent in Germany to 25 percent in Sweden (Table 1). In the country subsamples, the largest immigrant groups are from India (3%) and Pakistan (3%) in England, from Turkey (6%) and the former Soviet Union (4%) in Germany, from Turkey (2%) and Western Asia (2%) in the Netherlands, and from the former Yugoslavia (4%) and Turkey (2%) in Sweden.^1^

*Gender ideologies* of adolescents were assessed by asking respondents who they think should be responsible in a family for each of the tasks of cooking, cleaning, childcare, and earning money, respectively: mostly the woman, mostly the man, or both equally. As counter-stereotypical answers, e.g., women mostly earning money or men mostly cleaning, were chosen by very few respondents, we recoded the items into binary variables, differentiating between traditional respondents, who allocated unpaid work to women or paid work to men, and egalitarian individuals, who chose an equal division of labor or allocated unpaid work to men or paid work to women. With those categorical variables, we performed a polychoric factor analysis, creating a continuous scale from 0 (traditional) to 1 (egalitarian). *Cronbach's* α of 0.72 indicated acceptable reliability. As shown in Table 1, respondents had an average score of 0.75 units. Across subgroups, boys from both immigrant (0.61) and non-immigrant backgrounds (0.65) were more traditional than girls from immigrant (0.79) and non-immigrant backgrounds (0.86). To facilitate interpretation, we used a z-standardized version of this variable in our regression models.

To capture *educational aspirations*, respondents were asked to report the highest level of education they hoped to attain. We used the CILS4EU harmonized variable, which consisted of three categories: no degree or a degree below upper secondary school (1), an upper secondary school degree (2), and a university degree (3). In the full sample, most adolescents aimed at a university degree (65%), followed by an upper secondary school degree (25%; Table 1). Only 11% aspired to educational qualifications below an upper secondary school degree. This pattern persisted across gender and immigrant origin subgroups. However, descriptive results showed that young people from immigrant backgrounds had significantly higher aspirations, particularly girls (71% of boys and 79% of girls from immigrant backgrounds aspired to a university degree). Among non-immigrant adolescents, girls had higher aspirations as well (68% compared to only 55% for boys).

Information on adolescents' work values in relation to gender-typical occupational aspirations was elicited through the question “How important to you are the following aspects of a future occupation?” and three options: having a *high income, helping people*, and *thinking and solving problems*. Respondents answered on a scale from 1 (very important) to 4 (not at all important), which has been reversed to have higher values indicate a higher importance placed on the respective work value. In the full sample, having a high income appeared to be valued most strongly, with an average score of 3.16 units, followed by thinking and solving problems (3.13) and helping others (3.08; Table 1). Across subgroups, descriptive analyses and significance tests showed that adolescents from immigrant backgrounds gave significantly higher scores to all three items than their majority peers: valuing high income, helping others (within same-gender subgroups), and thinking and solving problems. In line with previous evidence, girls generally valued helping others more than boys, whereas boys valued a high income more. Thinking and solving problems was valued more highly by boys compared to girls among students of non-immigrant origin, but the opposite was true among immigrant-origin youth (Table 1). Again, we used z-standardized versions of these variables in our regression models.

As a control variable, we included the *country* indicator. However, this was a 6-fold variable, because observations from Germany and the Netherlands were further divided on the basis of information on academic vs. non-academic school tracks. This additional distinction is important, because whereas in England and Sweden all students remained in comprehensive general education, those in the Netherlands and Germany had already been tracked into different branches of the education system, and aspirations among students in the vocational training track may be considered as more realistic, as they probably already reflect the actual accessibility of different career choices. The six categories were: England (1), non-academic track in Germany (2), academic track in Germany (3), non-academic track in the Netherlands (4), academic track in the Netherlands (5), and Sweden (6). After weighting, 22% of respondents were in England, 27% in Germany, 26% in the Netherlands, and 25% in Sweden. Whereas in Germany, the non-academic and academic track subsamples had approximately the same size, the Dutch non-academic track was more than twice the size of the academic track (Table 1).

Lastly, we controlled for adolescents' socioeconomic background. Due to a lack of other information, we created a binary indicator based on *parents' educational level*, differentiating between adolescents whose parents had no tertiary education (0) and those who had at least one parent with a university degree (1). In the full sample, 40% of respondents came from families with at least one tertiary degree. Across subgroups, girls came from more highly educated backgrounds than boys, with girls from immigrant backgrounds being most likely to have at least one parent with tertiary education (44%). Further analyses show that this overrepresentation of immigrant girls from more highly educated backgrounds was driven by the English and German samples (not shown).

### 4.3. Analytical strategy

To model the probability of aspiring to occupations categorized as masculine (*m)*, feminine (*f* ), and ultra-feminine (*uf* ) compared to integrated (*i*), we estimated multinomial logistic regression models. We first ran a baseline model that only included the main independent subgroup variable, distinguishing girls and boys from immigrant and non-immigrant backgrounds (*g*), and the control variables (*c;* Equation 1).


(1) $$\begin{array}{l} \mathrm{Ln}\left(\frac{P\left(m/f/uf\right)}{P\left(i\right)}\right)=\beta_{1}+\beta_{2}g+\beta_{3}c \end{array}$$

In presenting the first set of results, we report predictive margins, which allowed for assessing intersectional subgroup differences by gender and immigrant background.

In a second step, we estimated an augmented model, which included gender ideologies (*gi*), educational aspirations (*ea*), valuing income (*vi*), valuing helping others (*vh*), and valuing thinking and problem solving (*vt*; Equation 2).


(2) $$\begin{array}{l} \begin{array}{lll} Ln(\frac{P(m/f/uf)}{P(i)}) & = & \beta_{1}+\beta_{2}g+\;\beta_{3}gi+\beta_{4}ea+\beta_{4}vi\;+\;\beta_{5}vh \end{array}\\ \;\begin{array}{ll} + & \;\beta_{6}vt\;+\beta_{7}c \end{array} \end{array}$$

Our goal in doing so was 2 fold. First, we aimed at understanding the pattern of associations between these mediators and the gender typicality of occupational aspirations. In presenting this set of results, we report average marginal effects, which allow assessing the contributions of different factors. Second, we wanted to investigate whether those values and beliefs actually explained or suppressed differences in occupational aspirations among boys and girls from immigrant and non-immigrant backgrounds by comparing subgroup coefficients in the specifications excluding and including gender ideology, educational aspirations, and the three distinct work values (Equation 1 vs. Equation 2). To ensure the comparability of coefficients across multinomial logistic models, we apply a correction proposed by Kohler et al. (2011) and refer to it as ‘KHB method'. In presenting the results, we report the coefficients and comment on each mediator's share of the overall effect obtained by disentangling each individual mediator's contribution (see also Karlson et al., 2012).

The last step of the analysis included interactions to test whether the associations between occupational aspirations and gender ideology, educational aspirations, and work values vary across groups.

The analyses were conducted using STATA 16. Throughout the analysis, we used a combination of design and adjustment weights provided by CILS4EU, which account for sample selection and non-response, correct the oversampling of immigrant-origin students, and assign the same contribution to each country (CILS4E, 2016). We ruled out multicollinearity issues among our independent variables by calculating variance inflation factors.

## 5. Results

We start by presenting results concerning the gender typicality of occupational aspirations across intersectional subgroups by gender and immigrant background. The following subsection examines the role of gender ideologies, educational aspirations, valuing income, valuing helping others, and valuing thinking and problem solving. The last subsection reports differential effects across subgroups.

### 5.1. Variations in gender typicality of occupational aspirations by gender and immigrant background

**Figure 2:**
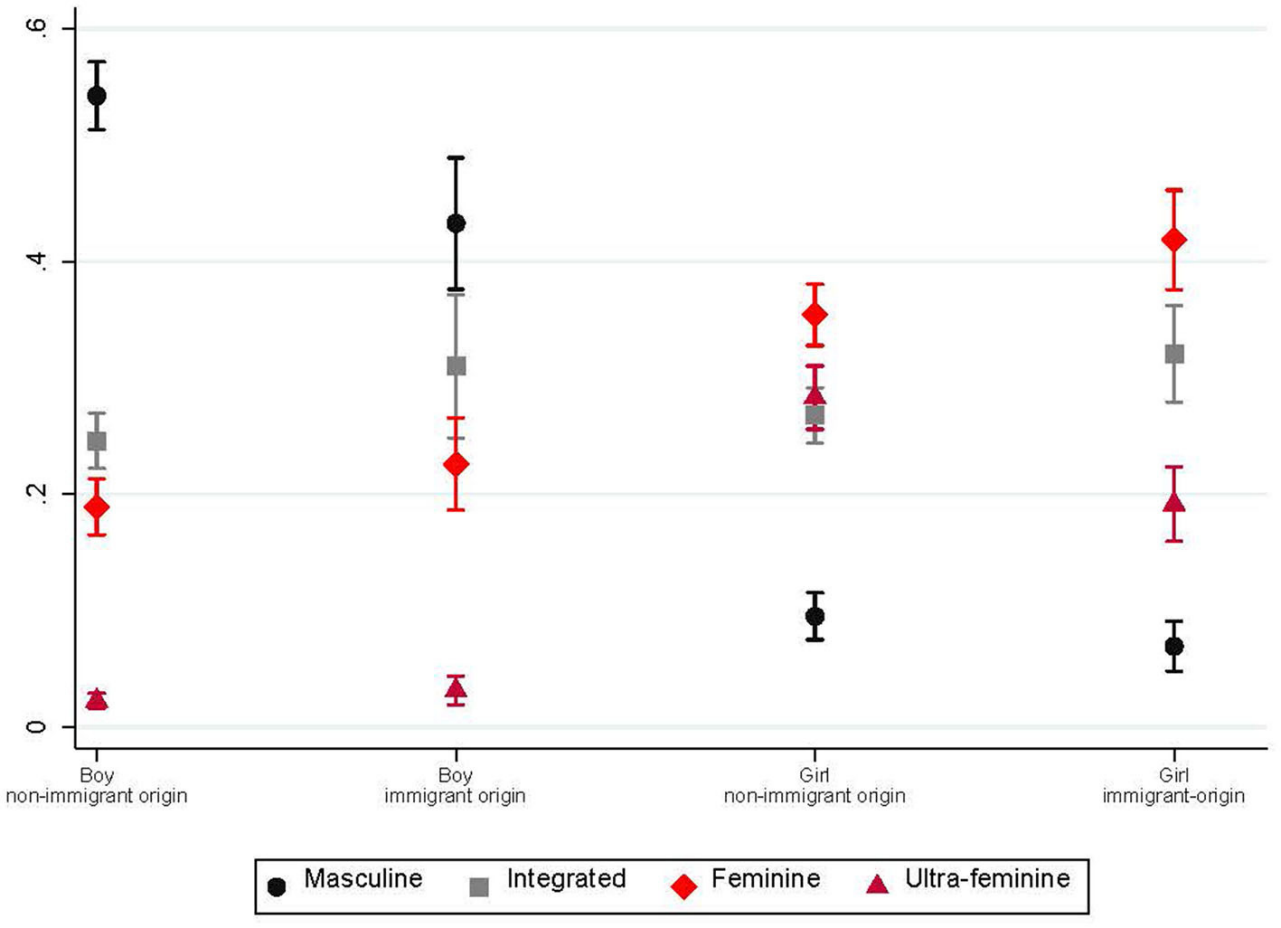
Occupational aspirations by gender and immigrant background group. Predictive margins of reduced multinomial logistic regression model of occupational aspirations on gender and immigrant background, additional control variables: parental education and country; pooled sample.

We start by exploring whether immigrant-origin adolescents have less gender-typical aspirations than their majority peers (Hypothesis 1a) and whether this difference is more pronounced among girls (Hypothesis 1b). Figure 2 and Table 2 report the predictive margins from the multinomial logistic regression model for the gender typicality of the aspired occupation, including only the country and school track indicator and parental education as control variables. As expected, boys and girls generally differed greatly in their aspirations. When estimating predictive margins, leaving aside the distinction by immigrant background, 52% of boys aspired to masculine occupations, compared to 9% of their female peers. Girls, on the other hand, mostly aspired to feminine (37%) and ultra-feminine occupations (27%), whereas only 20 and 2% of boys did so, respectively (Table 2).

**Table 2:** Predictive margins of occupational aspirations by subgroup estimated after the reduced multinomial logistic regression model including only control variables.

	**Masculine**	**Gender-balanced**	**Feminine**	**Ultra-Feminine**
Boys	0.523	0.257	0.196	0.024
Girls	0.091	0.277	0.365	0.267
Significance of contrasts of predictive margins	^***^	-	^***^	^***^
Non-immigrant boys	0.542	0.246	0.189	0.022
Immigrant-origin boys	0.433	0.310	0.226	0.031
Significance of contrasts of predictive margins	^***^	-	-	-
Non-immigrant girls	0.095	0.268	0.354	0.283
Immigrant-origin girls	0.069	0.321	0.419	0.191
Significance of contrasts of predictive margins	-	^*^	^*^	^***^
*N*	8,319	8,319	8,319	8,319

Control variables: parental education, country pooled sample. ^a^Significance of marginal contrasts between subgroups that were additionally calculated.

^*^p < 0.05, ^***^p < 0.001.

Immigrant-origin boys were significantly less likely to aspire to masculine occupations than boys of non-immigrant origin, with a difference of 11 percentage points (43 vs. 54%), whereas the latter were slightly more likely to aspire to integrated occupations (31 vs. 25%; *p* = 0.058). The probability of aspiring to (ultra)feminine occupations was fairly similar across the two groups without significant differences.

The occupational aspirations of girls showed substantial and significant differences between those of immigrant and non-immigrant origin for all categories of occupational aspirations except masculine occupations. Immigrant-origin girls were 5 percentage points more likely to aspire to integrated occupations (32 vs. 27%). Whereas immigrant-origin girls were also 6 percentage points more likely to aspire to feminine occupations (42 vs. 35%), they were far less likely than non-immigrant girls to aspire to ultra-feminine occupations, with a significant difference of 9 percentage points (28 vs. 19%). Overall, the first set of results were in line with Hypothesis 1a, which assumed that immigrant-origin adolescents would have less gender-typical occupational aspirations than their non-immigrant peers. We also found support that differences in gender typicality between immigrant and non-immigrant origin adolescents were larger among girls (Hypothesis 1b).

### 5.2. The role of gender ideology, educational aspirations, and work values

In a next step, our analyses explore the role of gender ideology, educational aspirations, and work values in predicting the gender typicality of occupational aspirations. Further, we examine whether these factors are potential mediators or suppressors of the differences in occupational aspirations among girls and boys from non-immigrant and immigrant backgrounds. Table 3 reports the average marginal effects of multinomial logistic regression models with the additional inclusion of gender ideology, educational aspirations, valuing income, valuing helping others, and valuing thinking and solving problems.

**Table 3:** Average marginal effects of full multinomial logistic regression model of occupational aspirations (weighted sample).

	**Masculine**	**Integrated**	**Feminine**	**Ultra-Feminine**
Immigrant-origin boys^a^	−0.067^*^	0.037	0.020	0.009
Non-immigrant girls^a^	−0.413^***^	0.027	0.134^***^	0.252^***^
Immigrant-origin girls^a^	−0.434^***^	0.075^**^	0.192^***^	0.167^***^
Gender ideologies	0.001	0.012	0.011	−0.024^***^
EA: Secondary school degree^b^	−0.043	0.071^**^	0.045	−0.072^**^
EA: University degree^b^	−0.169^***^	0.165^***^	0.136^***^	−0.131^***^
WV: High income	−0.003	0.028^***^	−0.013	−0.012
WV: Help others	−0.029^***^	−0.043^***^	0.018^*^	0.054^***^
WV: Think and solve problems	0.025^**^	0.004	−0.013	−0.016^**^
Germany (non-academic)^c^	0.019	−0.058^*^	−0.007	0.046^**^
Germany (academic) ^c^	0.014	−0.014	0.007	−0.006
Netherlands (non-academic)^c^	0.049	−0.061	−0.077^*^	0.090^***^
Netherlands (academic)^c^	−0.062^*^	−0.034	0.063^*^	0.033
Sweden^c^	0.055^**^	−0.074^***^	−0.050^**^	0.068^***^
Parents with tertiary education	−0.028	0.053^***^	0.015	−0.040^**^
*N*	8,319	8,319	8,319	8,319

EA, educational aspirations; WV, work values; ^a^Ref.: Non-immigrant boys. ^b^Ref.: Below secondary school degree or no degree. ^c^Ref.: England. ^*^p < 0.05, ^**^p < 0.01, ^***^p < 0.001.

First, we expected adolescents who are more egalitarian (Hypothesis 2a) and aim higher in education (Hypothesis 2b) to aspire to less gender-typical occupations. Furthermore, we hypothesized that adolescents who value a high income and thinking and solving problems more and helping others less aspire less to (ultra-)feminine occupations (Hypothesis 2c).

There was some support for Hypothesis 2a, but in relation to ultra-feminine occupations only. Indeed, stronger gender egalitarianism, equal to an increase of one standard deviation, was associated with a 2 percentage point decrease in adolescents' likelihood of aspiring to an ultra-feminine occupation.

Aiming high in education was associated with occupational aspirations away from both masculine and ultra-feminine occupations toward feminine and integrated occupations. As shown in Table 3, aspiring to a secondary school degree compared to no degree or less than a secondary school degree was associated with a decreased likelihood of aspiring to ultra-feminine occupations of 7 percentage points. Aspiring to a university degree compared to no degree or a degree below secondary school was related to a decreased likelihood of aspiring to both masculine (17 percentage points) and ultra-feminine occupations (13 percentage points). These associations aligned with the prediction that adolescents with higher educational aspirations would be less gender-typical in their occupational aspirations (Hypothesis 2b). However, contrary to Hypothesis 2b, students aspiring to a university degree were also 14-percentage points more likely to aspire to a feminine occupation compared to their peers aspiring to an upper secondary school degree or less.

Valuing income was associated with a lower propensity to aspire to any type of occupation other than the integrated one, but the effect did not reach statistical significance once educational aspirations were considered. Valuing thinking and solving problems was negatively associated with ultra-feminine occupations (2 percentage points per one standard-deviation increase) and positively associated with masculine occupations (3 percentage points) in the full model. The results therefore lent partial support to Hypothesis 2c, which assumed that valuing a high income and thinking and solving problems would contribute to orientating adolescents toward masculine occupations and away from (ultra-) feminine aspirations. Lastly, valuing helping others was significantly associated with a higher likelihood of aspiring to both feminine (2 percentage points per standard deviation) and ultra-feminine occupations (5 percentage points) and away from masculine occupations (3 percentage points), in line with Hypothesis 2d.

So far, we have established the direction and magnitude of the associations between the gender typicality of aspirations on the one hand and gender ideology, educational aspirations, and three work values on the other. We now examine to what extent these value orientations explain or suppress differences in occupational aspirations among boys and girls from non-immigrant and immigrant backgrounds. Are the differences reported in Figure 2 and Table 2, partly mediated or suppressed by underlying differences in values and beliefs? To answer this, we compared the coefficients of the different groups in a reduced model to the coefficients of augmented models that include the potential mediators. We applied the KHB method (Karlson et al., 2012) to ensure the comparability of coefficients. Tables 4, 5 present multilogit coefficients with integrated occupations as base outcome and using non-immigrant boys (Table 4) and non-immigrant girls (Table 5) as the respective reference categories for the intersection of gender and immigrant background. Each table compares the coefficients of the subgroups across six additional models, five including each mediator separately and one with all mediators together. The starting point are the coefficients of each subgroup obtained from the reduced model (first line of each panel). In the lines below, coefficients highlighted in bold are the ones that are statistically significantly different from the reduce model coefficient. So, for example, gender ideology, valuing problem solving and helping others do not mediate the lower likelihood of immigrant-origin boys to aspire to masculine occupations (Table 4, top panel). For a more accurate picture of the mediation effects, we disentangled the contribution of each mediator to the indirect as well as the overall effect (Tables 6, 7).

**Table 4:** Comparing subgroup coefficients of the reduced multinomial model including only control variables vs. models that additionally include gender ideology, educational aspirations, and work values (ref.: non-immigrant boys).

	**Masculine**	**Feminine**	**Ultra-feminine**
**Immigrant-origin boys**			
Reduced model	−0.474^**^	−0.053	0.091
	(−2.67)	(−0.29)	(0.31)
Incl. gender ideology	−0.480^**^	−0.050	0.058
	(−2.69)	(−0.27)	(0.20)
Incl. educational aspirations	**−0.312**	−0.042	**0.251**
	**(−1.69)**	(−0.23)	**(0.81)**
Incl. WV: high income	**−0.429** ^*^	**−0.005**	**0.165**
	**(−2.44)**	**(−0.03)**	**(0.55)**
Incl. WV: helping others	−0.478^**^	**−0.099**	**−0.050**
	(−2.68)	**(−0.54)**	**(−0.17)**
Incl. WV: thinking/solving problems	−0.485^**^	−0.055	0.078
	(−2.72)	(−0.30)	(0.26)
Incl. all mediators	**−0.314**	−0.038	**0.229**
	**(−1.69)**	(−0.20)	**(0.74)**
**Non-immigrant girls**			
Reduced model	−1.835^***^	0.548^***^	2.471^***^
	(−12.56)	(4.75)	(13.11)
Incl. gender ideology	−1.812^***^	0.538^***^	**2.600** ^***^
	(−12.31)	(4.41)	**(13.10)**
Incl. educational aspirations	**−1.754** ^***^	0.556^***^	**2.547** ^***^
	**(−11.62)**	(4.81)	**(13.07)**
Incl. WV: high income	−1.868^***^	**0.512** ^***^	**2.408** ^***^
	(−12.66)	**(4.39)**	**(12.76)**
Incl. WV: helping others	−1.845^***^	**0.479** ^***^	**2.284** ^***^
	(−12.72)	**(4.08)**	**(12.13)**
Incl. WV: thinking/solving problems	−1.833^***^	0.549^***^	2.459^***^
	(−12.56)	(4.76)	(13.09)
Incl. all mediators	**−1.747** ^***^	0.544^***^	**2.665** ^***^
	**(−11.51)**	(4.48)	**(12.96)**
**Immigrant-origin girls**			
Reduced model	−2.356^***^	0.532^***^	1.851^***^
	(−12.15)	(3.82)	(8.83)
Incl. gender ideology	−2.343^***^	0.526^***^	**1.924** ^***^
	(−12.10)	(3.79)	**(9.06)**
Incl. educational aspirations	–2.169^***^	0.548^***^	**2.054** ^***^
	**(**–**10.92)**	(3.92)	**(9.20)**
Incl. WV: high income	−2.342^***^	0.547^***^	1.871^***^
	(−12.09)	(3.96)	(8.87)
Incl. WV: helping others	−2.378^***^	**0.395** ^**^	**1.523** ^***^
	(−12.06)	**(2.82)**	**(7.06)**
Incl. WV: thinking/solving problems	−2.379^***^	0.528^***^	1.838^***^
	(−12.20)	(3.76)	(8.76)
Incl. all mediators	**−2.165** ^***^	0.542^***^	**2.113** ^***^
	**(−10.92)**	(3.89)	**(9.42)**
*N*	8,319	8,319	8,319

WV, work value. Ref.: Gender-balanced occupations. T-statistics in parentheses. Control variables: parental education, country. Asterisks refer to significance of coefficient (^*^p < 0.05, ^**^p < 0.01, ^***^p < 0.001). Bold lettering indicates a significant difference compared to the reduced model (*p* < 0.05).

**Table 5:** Comparing subgroup coefficients of the reduced multinomial model including only control variables vs. models that additionally include gender ideology, educational aspirations, and work values (ref.: non-immigrant girls).

	**Masculine**	**Feminine**	**Ultra-feminine**
**Non-immigrant boys**			
Reduced model	1.835^***^	−0.548^***^	−2.471^***^
	(12.56)	(-4.75)	(−13.11)
Incl. gender ideology	1.812^***^	−0.538^***^	**−2.600** ^***^
	(12.31)	(−4.41)	**(−13.10)**
Incl. educational aspirations	**1.754** ^***^	−0.556^***^	**−2.547** ^***^
	**(11.62)**	(−4.81)	**(−13.07)**
Incl. WV: high income	1.868^***^	**−0.512** ^***^	**−2.408** ^***^
	(12.66)	**(**–**4.39)**	**(−12.76)**
Incl. WV: helping others	1.845^***^	**−0.479** ^***^	**−2.284** ^***^
	(12.72)	**(-4.08)**	**(−12.13)**
Incl. WV: thinking/solving problems	1.833^***^	−0.549^***^	−2.459^***^
	(12.56)	(-4.76)	(−13.09)
Incl. all mediators	**1.747** ^***^	−0.544^***^	**−2.665** ^***^
	**(11.51)**	(−4.48)	**(−12.96)**
**Immigrant-origin boys**			
Reduced model	1.361^***^	−0.602^***^	−2.380^***^
	(6.73)	(−3.47)	(−9.17)
Incl. gender ideology	1.332^***^	−0.589^**^	**−2.541** ^***^
	(6.40)	(−3.24)	**(**–**9.37)**
Incl. educational aspirations	1.443^***^	−0.598^***^	−2.295^***^
	(6.76)	(−3.43)	(−8.50)
Incl. WV: high income	**1.439** ^***^	**−0.518** ^**^	**−2.243** ^***^
	**(7.08)**	**(−2.97)**	**(-8.57)**
Incl. WV: helping others	1.367^***^	**−0.578** ^***^	**−2.334** ^***^
	(6.76)	**(**–**3.30)**	**(**–**8.97)**
Incl. WV: thinking/solving problems	1.348^***^	−0.605^***^	−2.380^***^
	(6.65)	(−3.49)	(−9.09)
Incl. all mediators	1.433^***^	−0.582^**^	−2.436^***^
	(6.52)	(−3.17)	(−8.68)
**Immigrant-origin girls**			
Reduced model	−0.521^*^	−0.0163	−0.620^***^
	(−2.39)	(−0.13)	(−4.28)
Incl. gender ideology	−0.531^*^	−0.0119	**−0.675** ^***^
	(−2.43)	(−0.09)	**(**–**4.59)**
Incl. educational aspirations	**−0.414**	−0.00736	**−0.492** ^**^
	**(−1.84)**	(−0.06)	**(**–**3.28)**
Incl. WV: high income	**−0.473** ^*^	**0.0354**	**−0.537** ^***^
	**(−2.17)**	**(0.28)**	**(**–**3.65)**
Incl. WV: helping others	−0.533^*^	**−0.0837**	**−0.761** ^***^
	(−2.42)	**(−0.67)**	**(**–**5.07)**
Incl. WV: thinking/solving problems	−0.546^*^	−0.0212	−0.620^***^
	(−2.50)	(−0.17)	(−4.21)
Incl. all mediators	**−0.417**	−0.00127	−0.552^***^
	**(−1.85)**	(−0.01)	(−3.65)
*N*	8,319	8,319	8,319

WV, work value. Ref.: Gender-balanced occupations. T-statistics in parentheses. Control variables: parental education, country. Asterisks refer to significance of coefficient (^*^p < 0.05, ^**^p < 0.01, ^***^p < 0.001). Bold lettering indicates a significant difference compared to the reduced model (*p* < 0.05).

**Table 6:** Contribution of each mediator to the indirect and total effect (weighted sample; reference group: non-immigrant boys).

	**Masculine**		**Feminine**		**Ultra–feminine**	
	**Contribution (%) to:**		**Contribution (%) to:**		**Contribution (%) to:**	
	**Indirect effect**	**Total effect**	**Indirect effect**	**Total effect**	**Indirect effect**	**Total effect**
**Immigrant–origin boys**						
Gender ideology	−1.90	−0.76	−4.35	−2.51	−28.98	30.33
Educational aspirations	**85.58**	**34.21**	55.20	31.78	**154.92**	–**162.10**
WV: high income	**25.53**	**10.21**	**167.98**	**96.72**	**86.77**	**−90.80**
WV: help others	−0.02	−0.01	**−149.25**	**−85.94**	**−134.98**	**141.24**
WV: think/solve problems	−9.20	−3.68	30.42	17.52	22.27	−23.31
Total confounding effect	–	**39.97**	-	57.58	–	−104.64
**Non–immigrant girls**						
Gender ideology	14.50	0.98	−6.39	−1.48	**−239.36**	**−6.68**
Educational aspirations	**102.58**	**6.94**	−12.71	−2.95	**−200.58**	**−5.60**
WV: high income	−22.62	−1.53	**28.59**	**6.64**	**83.04**	**2.32**
WV: help others	−0.05	−0.00	**86.97**	**20.20**	**442.30**	**12.35**
WV: think/solve problems	5.58	0.38	3.55	0.82	14.61	0.41
Total confounding effect	-	6.76	-	**23.22**	-	2.79
**Immigrant-origin girls**						
Gender ideology	4.86	0.50	−5.78	−1.08	**−689.41**	**−6.10**
Educational aspirations	**100.66**	**10.45**	−33.65	−6.29	**−1693.15**	**−14.97**
WV: high income	6.65	0.69	−22.69	−4.24	−210.17	−1.86
WV: help others	−0.04	−0.00	**182.91**	**34.18**	**2,965.61**	**26.22**
WV: think/solve problems	−12.13	−1.26	−20.79	−3.88	−272.87	−2.41
Total confounding effect	-	**10.39**		**18.69**	-	0.88

Ref.: Integrated occupations. WV, work values. Positive values indicate mediation effects whereas negative values indicate suppressor effects. Bold lettering indicates a significant difference compared to the reduced model as shown in Table 3 (*p* < 0.05).

**Table 7:** Contribution of each mediator to the indirect and total effect (weighted sample; reference group: non-immigrant girls girls).

	**Masculine**		**Feminine**		**Ultra–feminine**	
	**Contribution (%) to:**		**Contribution (%) to:**		**Contribution (%) to:**	
	**Indirect effect**	**Total effect**	**Indirect effect**	**Total effect**	**Indirect effect**	**Total effect**
**Non–immigrant boys**						
Gender ideology	14.50	0.98	−6.39	−1.48	**−239.36**	**−6.68**
Educational aspirations	**102.58**	**6.94**	−12.71	−2.95	**−200.58**	**−5.60**
WV: high income	−22.62	−1.53	**28.59**	**6.64**	**83.04**	**2.32**
WV: help others	−0.05	−0.00	**86.97**	**20.20**	**442.30**	**12.35**
WV: think/solve problems	5.58	0.38	3.55	0.82	14.61	0.41
Total confounding effect	-	6.76	-	**23.22**	-	2.79
**Immigrant–origin boys**						
Gender ideology	−41.94	1.53	−5.96	−1.59	**−112.38**	**−8.25**
Educational aspirations	44.07	−1.60	1.76	0.47	13.99	1.03
WV: high income	**143.09**	**−5.20**	**58.29**	**15.51**	**85.29**	**6.26**
WV: help others	0.07	−0.00	**36.64**	**9.75**	**93.87**	**6.89**
WV: think/solve problems	−45.29	1.65	9.27	2.47	19.23	1.41
Total confounding effect	-	−3.64	-	**26.61**	**-**	**7.34**
**Immigrant–origin girls**						
Gender ideology	−5.02	−1.16	−8.08	−5.60	**−105.11**	**−8.24**
Educational aspirations	**98.68**	**22.71**	44.65	30.95	**244.69**	**19.17**
WV: high income	**36.62**	**8.43**	**169.02**	**117.15**	**170.50**	**13.36**
WV: help others	−0.03	−0.01	**−175.79**	**−121.83**	**−310.46**	**−24.33**
WV: think/solve problems	−30.26	−6.96	70.20	48.65	100.37	7.86
Total confounding effect	-	**23.02**	-	69.31	-	7.84

Ref.: Integrated occupations. WV, work values. Positive values indicate mediation effects whereas negative values indicate suppressor effects. Bold lettering indicates a significant difference compared to the reduced model as shown in Table 4 (*p* < 0.05).

We expected higher educational aspirations and valuing a high income to partly explain the less gender-typical occupational aspirations among immigrant-origin adolescents compared to their non-immigrant peers (Hypothesis 3a). In line with this hypothesis, our results showed that higher educational aspirations and valuing a high income were implicated in the differences between adolescents from non-immigrant and immigrant backgrounds. The bold coefficients of educational aspirations and high income in the first column of Table 4 indicate that they differ significantly from the coefficient of the reduced model and by being smaller in magnitude they imply that differences in educational aspirations and in the value attached to earning a high income are found to be significant mediators, partly explaining immigrant-origin boys' weaker preference for masculine occupations compared to non-immigrant boys. Likewise, the bold coefficients of educational aspirations and high income in the last column of Table 5 indicate that they mediated immigrant-origin girls' weaker preference for ultra-feminine occupations compared to non-immigrant girls.

Gender egalitarianism, on the other hand, was expected to suppress differences in the gender typicality of occupational aspirations between youth from immigrant vs. non-immigrant backgrounds (Hypothesis 3b). In line with this assumption, the inclusion of gender egalitarianism increased the difference in aspirations to ultra-feminine occupations between non-immigrant and immigrant-origin girls (Table 5), but did not alter the difference between boys from immigrant and non-immigrant backgrounds (Table 4).

When assessing the contributions of the mediators (Tables 6, 7), we found that, taken together, value orientations explained 40% of immigrant-origin boys' lesser propensity to aspire to masculine occupations relative to their male peers. And it was specifically educational aspirations which contributed the most, followed by valuing a high income. In other words, the lower gender typicality of the aspirations of boys of immigrant origin could be partly traced back to their ambitious educational aspirations and their valuing income more than their peers from non-immigrant backgrounds.

By contrast, among girls, the overall explanatory contribution of value orientations was lower because of the opposite effect of different individual value orientations. The lesser propensity of girls of immigrant origin to aspire to ultra-feminine occupations was largely due to their higher educational aspirations and their valuing a high income more than their female peers. At the same time, immigrant-origin girls' less egalitarian gender ideologies and stronger altruistic values suppressed those effects. As a result, only 8% of the overall difference between girls from immigrant and non-immigrant backgrounds in aspiring to ultra-feminine occupations was accounted for by the mediators. When looking at differences in aspirations to feminine occupations, a similar pattern emerged. Whereas valuing income contributed to the overall effect, valuing helping others acted as a suppressor, so that girls of immigrant origin's higher propensity to aspire to female occupations could not be neatly attributed to different sets of values relative to girls from non-immigrant backgrounds.

All in all, the group coefficients remained fairly stable across the augmented models and their statistical significance was never absorbed by the inclusion of any mediating factors, whether individually or combined. This is in line with a latent class analysis that we had originally conducted to identify profiles of value orientations, but which did not yield meaningful results, suggesting that gender ideology, educational aspirations, and different work values did not cluster in typical combinations.

### 5.3. Differential effects

**Figure 3:**
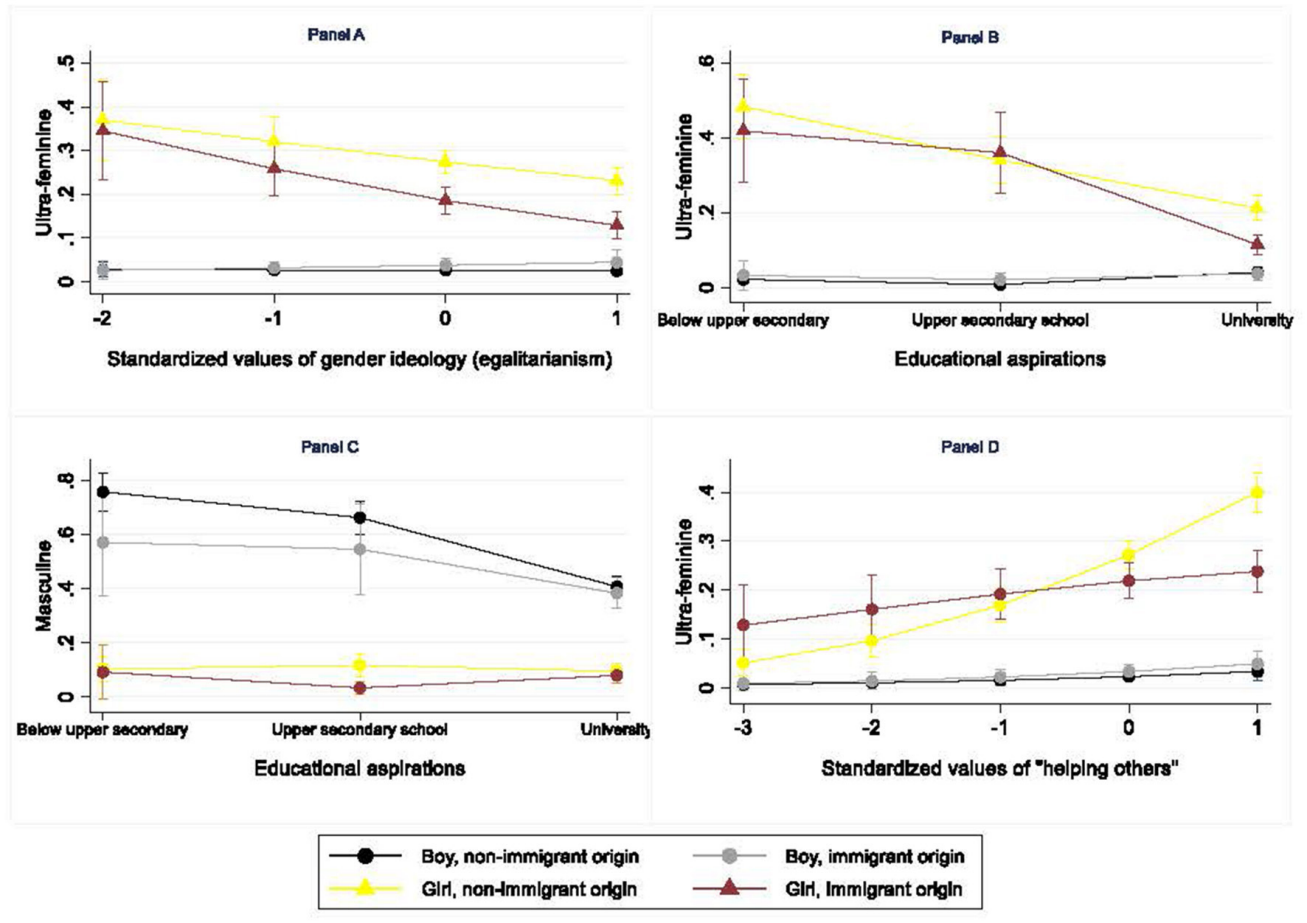
Aspirations by subgroup and gender ideology, educational aspirations, and work values. Predictive margins of aspiring to ultra-feminine occupations **(A, B, D)** or masculine occupations **(C)** derived from multinomial logistic regression model of occupational aspirations on gender and immigrant background interacted with gender ideology **(A)**, educational aspirations **(B, C)**, work-related value “helping others” **(D)**; all specifications include additional control variables: parental education and country; pooled sample.

In the last step of our analysis, we explore whether the associations between occupational aspirations on the one hand and gender ideology, educational aspirations, and work values on the other vary across groups. We reran our models including interactions with the subgroup variable and each of the main independent variables, respectively (Supplementary Tables S1, S2). Results revealed a few differential effects. First, Figure 3A shows that higher egalitarianism was associated with being less likely to aspire to ultra-feminine occupations among girls but not boys. If a non-immigrant (immigrant) girl's egalitarianism increased by one standard deviation from the mean, her likelihood of aspiring to an ultra-feminine occupation decreased by 4 (5) percentage points, while no such change is observed for boys.

Second, aiming high in education appeared to have consistently stronger effects on adolescents from immigrant backgrounds than on their peers. Aspiring to a university degree compared to a degree below upper secondary education was associated with a reduced likelihood of aspiring to ultra-feminine occupations by 27 and 31 percentage points among girls of non-immigrant and immigrant origins, respectively (Figure 3B). Among boys, educational aspirations influenced aspirations to masculine occupations, with much more pronounced effects among boys of immigrant origin. Whereas a boy of non-immigrant origin aspiring to a university degree was 19 percentage points less likely to aspire to a masculine occupation than a peer aiming below upper secondary education, the difference amounted to 35 percentage points among boys of immigrant origin (Figure 3C).

Third, among girls of non-immigrant origin, valuing “helping others” was more strongly associated with a higher likelihood of aspiring to ultra-feminine occupations than among other groups (Figure 3D). For them, an increase of one standard deviation in valuing helping others corresponded to a 13 percentage point higher probability of aspiring to an ultra-feminine occupation.

### 5.4. Cross-national variations

To examine whether the patterns presented applied to all countries or were driven by specific countries, we additionally ran all analyses with individual country subsamples (Supplementary Tables S3–S19), and, if applicable, used interactions between our independent variables and the country indicator in the full sample to test whether the differences were significant (not shown, available from the authors on request). The results indicated high similarity across countries, with some differences in effect sizes and significance but the same overall patterns. Here, we report those country differences that were significant or appeared to be driving the pooled sample effect, or if they pointed to effects that contradict previous findings in the full sample.

First, differences in occupational aspirations by gender and immigrant background were largely similar across countries (Hypotheses 1a and 1b, Supplementary Tables S3–S7). However, the differences between non-immigrant and immigrant-origin girls were particularly large in England: there, non-immigrant girls had a predicted probability of 22% of aspiring to ultra-feminine occupations, whereas among immigrant-origin girls, it was only 8%. Immigrant-origin girls in England also stood out because they were significantly more likely than non-immigrant girls to aspire to integrated occupations (29 vs. 46%; see Supplementary Figure S1, Supplementary Table S7). A possible explanation for England's distinctiveness lies in the very low prestige of ultra-feminine occupations and a labor market characterized by large earning differentials (Grimshaw and Rubery, 2007).

Second, the effects of values and beliefs also proved to be very similar across countries, with only minor differences (Hypotheses 2a, 2b, 2c, and 2d; Supplementary Tables S8–S11). Regarding potential mediating or suppressing effects of gender ideologies, educational aspirations and the three sets of work values (Hypothesis 2d), the results were also rather similar (Supplementary Tables S12–S19). The mediating role of educational aspirations was strongest in the England sample, where it explained immigrant-origin girls' much weaker preference for ultra-feminine occupations compared to non-immigrant girls. For Germany and the Netherlands, we additionally checked that the effect of educational aspirations was not absorbed by our controlling for educational track (not shown). Given the complex relationship between occupations educational entry requirements and gender segregation (Estévez-Abe, 2006), this finding does not necessarily contradict previous evidence on the role of educational aspirations in orientating adolescents toward higher status occupations.

Lastly, testing whether the associations between values and beliefs vary across groups revealed similar patterns, with one exception (Hypothesis 3). Only in the Netherlands did the association of valuing helping others with aspiring to ultra-feminine occupations not vary across groups (not shown).

## 6. Discussion and conclusion

This paper examined adolescents' occupational aspirations, assessing their gender typicality, as captured by the gender composition of the aspired occupation. The analyses employed CILS4EU data, which allowed for comparisons between adolescents of immigrant and non-immigrant origin in England, Germany, the Netherlands, and Sweden in 2012. We first assessed to what extent the gender typicality of aspirations differed between boys and girls from immigrant and non-immigrant backgrounds. Second, we explored whether gender ideology, work values and educational aspirations accounted for some of those differences.

In line with previous literature (e.g., Sikora and Saha, 2009; Polavieja and Platt, 2014; Barrett, 2021; Stoet and Geary, 2022), our results showed that adolescents' occupational aspirations were highly gendered, with girls being more likely to aspire to female-dominated occupations and boys to male-dominated occupations. Yet boys and girls of immigrant origin aspired to somewhat less gender-typical occupations than their majority peers. This finding concurs with a recent study on Germany by Wicht and Siembab (2022), which showed that students from immigrant backgrounds had less gender-typical occupational aspirations than natives on average, and the difference was especially pronounced for students from Turkey, which is one of the largest immigrant groups in our sample as well. Our findings also indicate that adolescents of immigrant origin orientated themselves toward more integrated occupations, as they were not more likely than their majority peers to aspire to gender-atypical occupations. Overall, the differences we detected were significant but not huge. Yet, given the persistency of gender typicality in occupational aspirations, these variations merit consideration and suggest that adolescents from immigrant backgrounds may be catalysts of some change in women's position on the labor market, despite their frequently more traditional gender ideologies.

Our analyses of possible mediators of these intersectional differences in the gender typicality of occupational aspirations suggest that higher gender egalitarianism partly accounts for variations only among girls by encouraging female adolescents to aspire to higher-status jobs and away from female-dominated occupations. However, they do not contribute to explaining differences by immigrant background nor differences among boys. These findings differ somewhat from previous studies (Lawson et al., 2018; Chesters, 2021), which found more egalitarian gender ideologies to relate to less gender-typical occupational aspirations and choices among both boys and girls in Germany and the US, respectively. van der Vleuten et al. (2016) found such a relationship with gender-typical subject choice only among boys in the Netherlands. The limited explanatory power of gender ideologies is, however, in line with Cech (2013), who found implicit gendered self-images to be more predictive of the gender typicality of occupational choices than gender ideologies.

By contrast, the less gender typical occupational aspirations of boys and girls from immigrant backgrounds compared to non-immigrant boys and girls were partly accounted for by their more ambitious educational aspirations, which orientated them away from low-prestige jobs. This finding extends the large literature on the relatively higher educational aspirations of immigrant-origin youth (Kristen and Dollmann, 2009; Jackson et al., 2012; Nauck and Schnoor, 2015; Hadjar and Scharf, 2019; Möser, 2022) by also linking them with less gender typical occupational aspirations. However, it also highlights the non-linear relationship between the vertical ordering of occupations, whether by qualification requirements, pay, or prestige, and their gender typicality. Indeed the mediating role of educational aspirations was most evident in England, a country characterized by large earnings differentials and where ultra-feminine occupations are often low-status and low-paid (Grimshaw and Rubery, 2007; Eurofound, 2017). In countries where female-dominated occupations enjoy higher prestige, it is credible that educational aspirations would not be such an important mediating factor (Wicht and Siembab, 2022). Remarkably, valuing a high income hardly contributed to explaining variations by gender and immigrant background once educational aspirations were accounted for. Our finding that girls attach greater value to helping others in their jobs confirms long-standing findings on women's preference for pro-social values (Marini et al., 1996; Busch-Heizmann, 2014). They help explain some of the gender differences in occupational aspirations, in line with work by Weisgram et al. (2010, 2011). In addition, we show that they also moderately contribute to explaining differences between girls from immigrant backgrounds and their non-immigrant peers with respect to the lower probability of aspiring to ultra-feminine occupations among the former compared to the latter. The explanatory relevance of valuing thinking and solving problems in one's job is less clear-cut, partly as we also find smaller differences along this dimension by gender and immigrant background.

Overall, our analyses that jointly consider gender ideologies, educational aspirations and different work values suggest that the combinations of beliefs and values held by adolescents are intricate and complex and often counteract each other. Interestingly, our study shows that adolescents of immigrant origin in the four countries attached slightly greater importance on average to earning a high income, helping others and to thinking and solving problems in their future jobs compared to their majority peers. Immigrant adolescents' simultaneous endorsement of many work values confirms that extrinsic and intrinsic work values are not necessarily in opposition (Kraaykamp et al., 2019), but also indicates that adolescents of immigrant origin assign higher value to education (Hadjar and Scharf, 2019) while attributing to their future job greater importance for fulling their personal values. This finding also applies to girls, as differences in gender typicality would be even larger if they were not suppressed by the more traditional gender ideologies held by girls of immigrant backgrounds. Overall, this may suggest that children of immigrant origin do not prioritize instrumental concerns more than those from non-immigrant backgrounds, possibly because the former have a stronger drive to self-actualize through their jobs, both in terms of material achievements and self-expression.

Our comparative analyses of four European countries with different labor market systems, levels of wage inequality and gender cultures is informative, as it shows mostly similar differences between youth of immigrant and non-immigrant origin in terms of occupational aspirations, educational aspirations, gender ideologies, and work values. The overall similarity across the four countries is in line with other comparative studies based on CILS4EU, which found variations in countries' education systems to be less important than expected in explaining the differences between adolescents from immigrant and non-immigrant backgrounds in educational aspirations (Hadjar and Scharf, 2019) and choices (Dollmann and Weißmann, 2020). The few distinctive findings we could uncover suggest that more in-depth comparisons of how the aspirations of youth before entering the labor market are influenced by the structural forces that shape occupations and their rewards might represent a promising line of investigation.

The exploratory nature of this study makes it all the more important to recognize its limitations, as they can highlight possible avenues for future research. Previous studies have shown that immigrant subgroups in the four countries vary markedly in their gender ideologies, educational and occupational aspirations (e.g., de Valk, 2008; Wicht and Siembab, 2022). It would therefore be insightful to conduct further analyses of specific groups, which were unfortunately beyond the scope of our study. Our categorical measures of occupations' gender typicality allowed for less variation than continuous measures, but arguably better reflected non-linearity. It remains the case that measures based on statistical classifications of occupations cannot tap into adolescents' perceptions of occupations and their affordances. Future research could engage more directly with adolescents' assessments of jobs to better understand their aspirations and choices and also the extent to which occupational classifications mirror their understanding of the labor market. Furthermore, data access restrictions to the full data, especially during the COVID-19 pandemic, limited the feasibility of using different measures of occupational aspirations.

Further data limitations include limited variation in educational aspirations and gender ideologies in some countries, especially Sweden. Recent research (Grunow and Veltkamp, 2016; Knight and Brinton, 2017) has argued that gender ideologies are better conceptualized as multidimensional, as individuals' beliefs regarding the appropriate division of paid work may differ from what they consider best in the domain of childcare and for children's wellbeing. Future large-scale panel studies should therefore include items tapping into different dimensions.

Ideally, we would have liked to draw on panel data to explore how the occupational aspirations, gender ideologies, educational aspirations, and work values of adolescents of immigrant and non-immigrants origin develop across adolescence and into early adulthood. Unfortunately, most of these measures were only available at one point in time in the CILS4EU data, making it also impossible to gain information on about 30 percent of the adolescents who did not indicate any occupational aspiration at age 16. Our cross-sectional analyses represent correlations and cannot be interpreted as hinting at causal relationships. This is important to acknowledge, as for instance, occupational aspirations and educational aspirations may be endogenous. High educational aspirations may pave the way for pursuing less female-dominated occupations but aspiring to specific occupations that require higher educational qualifications may also motivate students to aspire to higher levels of education. Finally, it is important to bear in mind that our analyses refer to occupations aspirations and that we cannot follow most of the students in the sample long enough to observe the extent to which occupational choices match aspirations.

Despite its limitations, this exploratory study advances our knowledge about adolescents' occupational aspirations in several ways. The findings document the intersectional patterns of gender typical occupational aspirations among girls and boys of immigrant and non-immigrant backgrounds in four wealthy European countries. The findings also point to the complex ways in which value orientations contribute to variations in gender typical aspirations. Overall, our approach and findings confirm the importance of distinguishing between multiple dimensions of adolescents' value orientations, covering not only educational aspirations but also gender ideology and values related to work, in order to better identify the mechanisms underlying their occupational aspirations.

## Data availability statement

The main dataset analyzed in this study is publicly available. The data is available at the GESIS data archive for the social science, Cologne, Germany. Study No. ZA5353, https://doi.org/10.4232/cils4eu.5353.3.3.0.

## Author contributions

LG: conceptualization (equal), formal analysis (supervision), writing—original draft preparation, and writing—review and editing (equal). JW: data curation, formal analysis, writing—original draft (supporting), and writing—review and editing (supporting). PS: funding acquisition, conceptualization (equal), formal analysis (supervision), writing—review and editing (equal), and supervision (lead). All authors contributed to the article and approved the submitted version.

## Footnotes

^1^We also used another definition of groups to assess whether differences in occupational aspirations were masked by some immigrant groups being very similar to those of non-immigrant origin. We identified Muslim adolescents and contrasted them to their non-religious or Christian peers, based on existing evidence of value differences coinciding with religious faith (Diehl et al., 2009). The results did not substantially differ from those presented here.

## References

[B1] Addati L. Cattaneo U. Esquivel V. Valarino I. (2018). Care Work and Care Jobs for the Future of Decent Work. Genève: ILO.

[B2] Barone C. (2011). Some things never change: gender segregation in higher education across eight nations and three decades. Sociol. Educ. 84, 157–176. 10.1177/0038040711402099

[B3] Barrett E. (2021). Career aspirations of teenagers and the future of gender equality in occupations. J. Educ. Work 34, 110–127. 10.1080/13639080.2021.1887829

[B4] Bettio F. Tinios P. Betti G. (2013). The Gender Gap in Pensions in the EU. Luxembourg: European Commission - Directorate-General for Justice.

[B5] Budig M. J. Misra J. (2010). How care-work employment shapes earnings in cross-national perspective. Int. Labour Rev. 149, 441–460. 10.1111/j.1564-913X.2010.00097.x

[B6] Busch-Heizmann A. (2014). Supply-side explanations for occupational gender segregation: adolescents' work values and gender-(A)Typical Occupational Aspirations. Eur. Sociol. Rev. 31, 48–64. 10.1093/esr/jcu081

[B7] Bussey K. Bandura A. (1999). Social cognitive theory of gender development and differentiation. Psychol. Rev. 106, 676–713. 10.1037/0033-295X.106.4.676 10560326

[B8] Cech E. A. (2013). The self-expressive edge of occupational sex segregation. Am. J. Sociol. 119, 747–789. 10.1086/673969

[B9] Cemalcilar Z. Secinti E. Sumer N. (2018). Intergenerational transmission of work values: a meta-analytic review. J. Youth Adolesc. 47, 1559–1579. 10.1007/s10964-018-0858-x 29744707

[B10] Chang M. L. (2000). The Evolution of Sex Segregation Regimes. Am. J. Sociol. 105, 1658–1701. 10.1086/210469

[B11] Charles M. (2017). Venus, mars, and math: Gender, societal affluence, and eighth graders' aspirations for STEM. Socius. 3. 10.1177/2378023117697179

[B12] Charles M. Bradley K. (2009). Indulging our gendered selves? Sex segregation by field of study in 44 countries. AJS. 114, 924–976. 10.1086/595942 19824299

[B13] Charles M. Grusky D. B. (2004). Occupational Ghettos: The Worldwide Segregation of Women and Men. Stanford University Press. Available online at: https://books.google.de/books?id=qqIEAQAAIAAJ

[B14] Chesters J. (2021). Gender Attitudes and Occupational Aspirations in Germany: Are Young Men Prepared for the Jobs of the Future? Work, Employ. Soc. 37, 571–587. 10.1177/09500170211017046

[B15] Chesters J. (2022). Understanding the Persistence of Occupational Sex Segregation in German Labour Markets: How Gender Attitudes Shape Young Women's Occupational Aspirations. J. Appl. Youth Stud. 5, 55–73. 10.1007/s43151-021-00065-1

[B16] Chung H. (2019). ‘Women's work penalty' in access to flexible working arrangements across Europe. Eur. J. Ind. Relat. 25, 23–40. 10.1177/0959680117752829

[B17] CILS4E (2016). Children of Immigrants Longitudinal Survey in Four European Countries. Technical Report. Wave 2 – 2011/2012, v2.3.0; Mannheim: Mannheim University.

[B18] Correll S. J. (2001). Gender and the career choice process: the role of biased self-assessments. Am. J. Sociol. 106, 1691–1730. 10.1086/321299

[B19] Correll S. J. (2004). Constraints into preferences: gender, status, and emerging career aspirations. Am. Sociol. Rev. 69, 93–113. 10.1177/000312240406900106

[B20] Davis S. N. Greenstein T. N. (2009). Gender ideology: Components, predictors, and consequences. Ann. Rev. Sociol. 35, 87–105. 10.1146/annurev-soc-070308-115920

[B21] de Valk H. A. G. (2008). Parental influence on work and family plans of adolescents of different ethnic backgrounds in The Netherlands. Sex Roles 59, 738–751. 10.1007/s11199-008-9464-927080996

[B22] Diehl C. Koenig M. Ruckdeschel K. (2009). Religiosity and gender equality: comparing natives and Muslim migrants in Germany. Ethnic Racial Stud. 32, 278–301. 10.1080/01419870802298454

[B23] Dollmann J. (2017). Positive choices for all? SES- and gender-specific premia of immigrants at educational transitions. Res. Soc. Stratif. Mobil. 49, 20–31. 10.1016/j.rssm.2017.03.001

[B24] Dollmann J. Weißmann M. (2020). The story after immigrants' ambitious educational choices: real improvement or back to square one? Eur. Sociol. Rev. 36, 32–47. 10.1093/esr/jcz042

[B25] Drouhot L. G. Nee V. (2019). Assimilation and the Second Generation in Europe and America: blending and segregating social dynamics between immigrants and natives. Ann. Rev. Sociol. 45, 177–199. 10.1146/annurev-soc-073117-041335

[B26] Eccles J. S. Wigfield A. (2002). Motivational beliefs, values, and goals. Annu. Rev. Psychol. 53, 109–132. 10.1146/annurev.psych.53.100901.135153 11752481

[B27] England P. (2010). The gender revolution: uneven and stalled. Gender Soc. 24, 149–166. 10.1177/0891243210361475

[B28] England P. Allison P. Wu Y. (2007). Does bad pay cause occupations to feminize, Does feminization reduce pay, and How can we tell with longitudinal data? Soc. Sci. Res. 36, 1237–1256. 10.1016/j.ssresearch.2006.08.003 10.1016/j.ssresearch.2006.08.003

[B29] England P. Levine A. Mishel E. (2020). Progress toward gender equality in the United States has slowed or stalled. Proc. Natl. Acad. Sci. U S A. 117, 6990–6997. 10.1073/pnas.1918891117 32229559 PMC7132302

[B30] Estévez-Abe M. (2006). Gendering the varieties of capitalism: A study of occupational segregation by sex in advanced industrial societies. World Polit. 59, 142–175. 10.1353/wp.2007.0016

[B31] Eurofound (2017). Occupational change and wage inequality: European Jobs Monitor 2017. Publications Office of the European Union. Available online at: https://www.eurofound.europa.eu/sites/default/files/ef_publication/field_ef_document/ef1710en.pdf (accessed June 14, 2023).

[B32] Eurostat (2020). EU - Labour Force Survey microdata 1983-2019 Version version 1.

[B33] Gallie D. (2019). Research on work values in a changing economic and social context. Ann. Am. Acad Polit. Soc. Sci. 682, 26–42. 10.1177/0002716219826038

[B34] García-Mainar I. Montuenga V. M. García-Martín G. (2018). Occupational prestige and gender-occupational segregation. Work, Employm. Soc. 32, 348–367. 10.1177/0950017017730528

[B35] Gautié J. Schmitt J. (2010). Low-Wage Work in the Wealthy World. New York: Russell Sage Foundation.

[B36] Gerber T. P. Cheung S. Y. (2008). Horizontal Stratification in Postsecondary Education: Forms, Explanations, and Implications. Ann. Rev. Sociol. 34, 299–318. 10.1146/annurev.soc.34.040507.134604

[B37] Glass J. Fujimoto T. (1995). Employer characteristics and the provision of family responsive policies. Work Occup. 22, 380–411. 10.1177/0730888495022004002

[B38] Glauber R. (2011). LIMITED ACCESS: gender, occupational composition, and flexible work scheduling. Sociol. Quart. 52, 472–494. 10.1111/j.1533-8525.2011.01215.x 22081800

[B39] Goldscheider F. Goldscheider C. Bernhardt E. M. (2011). Creating egalitarian families among the adult children of turkish- and polish-origin immigrants in Sweden. Int. Migr. Rev. 45, 68–88. 10.1111/j.1747-7379.2010.00839.x 21717599

[B40] Gottfredson L. S. (1981). Circumscription and compromise: A developmental theory of occupational aspirations. J. Counsel. Psychol. 28, 545–579. 10.1037/0022-0167.28.6.545

[B41] Grimshaw D. Rubery J. (2007). Undervaluing women's work. (Vol. Equal Opportunities Commission Working Paper Series no.53). Equal Opportunities Commission.

[B42] Grönlund A. Magnusson C. (2013). Devaluation, crowding or skill specificity? Exploring the mechanisms behind the lower wages in female professions. Soc. Sci. Res. 42, 1006–1017. 10.1016/j.ssresearch.2013.03.001 23721670

[B43] Grunow D. Evertsson M. (2016). Couples' Transitions to Parenthood. Cheltenham: Edward Elgar. 10.4337/9781785366000

[B44] Grunow D. Veltkamp G. (2016). “Institutions as reference points for parents-to-be in European societies: a theoretical and analytical framework,” in Couples' Transitions to Parenthood: Analysing Gender and Work in Europe, eds. D., Grunow, and M., Evertsson (Cheltenham: Edward Elgar). 10.4337/9781785366000.00009

[B45] Gutman L. M. Schoon I. Sabates R. (2012). Uncertain aspirations for continuing in education: antecedents and associated outcomes. Dev. Psychol. 48, 1707–1718. 10.1037/a0026547 22182295

[B46] Haasler S. R. Gottschall K. (2015). Still a perfect model? The gender impact of vocational training in Germany. J. Vocat. Educ. Train. 67, 78–92. 10.1080/13636820.2014.922118

[B47] Hadjar A. Scharf J. (2019). The value of education among immigrants and non-immigrants and how this translates into educational aspirations: a comparison of four European countries. J. Ethnic Migr. Stud. 45, 711–734. 10.1080/1369183X.2018.1433025

[B48] Hakim C. (1993). Segregated and integrated occupations: A new approach to analysing social change. Euro. Sociol. Rev. 9, 289–314. 10.1093/oxfordjournals.esr.a036682

[B49] Hayes A. R. Bigler R. S. Weisgram E. S. (2018). Of men and money: characteristics of occupations that affect the gender differentiation of children's occupational interests. Sex Roles 78, 775–788. 10.1007/s11199-017-0846-8

[B50] Heinrich-Böll-Stiftung (2010). Bis in die dritte Generation? Lebensrealitäten junger MigrantInnen. [Up to third generation? Life realities of young migrants]. Available online at: https://heimatkunde.boell.de/sites/default/files/dossier_dritte_generation.pdf (accessed June 14, 2023).

[B51] Idema H. Phalet K. (2007). Transmission of gender-role values in Turkish-German migrant families: The role of gender, intergenerational and intercultural Relations. Zeitschrift Für Familienforschung. 19, 71–105. 10.20377/jfr-338

[B52] ILO (2012). International Standard Classification of Occupations 2008 - Structure, Group Definitions and Correspondence Tables. In (Vol. 1). Geneva: ILO.

[B53] Jackson M. Jonsson J. O. Rudolphi F. (2012). Ethnic Inequality in Choice-driven Education Systems:A Longitudinal Study of Performance and Choice in England and Sweden. Sociol. Educ. 85, 158–178. 10.1177/003804071142731133960395

[B54] Johnson M. K. (2001). Change in job values during the transition to adulthood. Work Occupat. 28, 315–345. 10.1177/0730888401028003004

[B55] Johnson M. K. Mortimer J. T. (2011). Origins and outcomes of judgments about work. Soc. Forces 89, 1239–1260. 10.1093/sf/89.4.1239 21765555 PMC3134872

[B56] Joppke C. (2007). Transformation of immigrant integration: Civic Integration and Antidiscrimination in the Netherlands, France, and Germany. World Politics 59, 243–273. 10.1353/wp.2007.0022

[B57] Kalter F. Heath A. Hewstone M. Jonsson J. Kalmijn M. Kogan I. I.. (2016a). Children of Immigrants Longitudinal Survey in Four European Countries (CILS4EU) – Reduced version. Reduced data file for download and off-site use., ZA5656 Data file Version 1.2.0, GESIS Data Archive, Cologne.

[B58] Kalter F. Heath A. Hewstone M. Jonsson J. Kalmijn M. Kogan I. I.. (2016b). Children of Immigrants Longitudinal Survey in Four European Countries (CILS4EU) - Reduced version. Reduced data file for download and off-site use. Version ZA5656 Data file Version 2.3.0, GESIS Data Archive.

[B59] Karlson K. B. Holm A. Breen R. (2012). Comparing regression coefficients between same-sample nested models using logit and probit: A new method. Sociol. Methodol. 42, 286–313. 10.1177/0081175012444861

[B60] Knight C. R. Brinton M. C. (2017). One egalitarianism or several? Two decades of gender-role attitude change in Europe. Am. J. Sociol. 122, 1485–1532. 10.1086/689814

[B61] Kogan I. (2018). Ethnic Minority Youth at the Crossroads: Between Traditionalism and Liberal Value Orientations,” in Growing Up in Diverse Societies. The Integration of the Children of Immigrants in England, Germany, the Netherlands and Sweden, eds. F., Kalter, J. O. Jonsson, F., van Tubergen, and A., Heath (Oxford: Oxford University Press) 303–331. 10.5871/bacad/9780197266373.003.0012

[B62] Kohlberg L. (1969). “Stage and sequence: The cognitive developmental approach to socialization,” in Handbook of Socialization Theory and Research, ed D. Goslin (Chicago, IL: Rand McNally), 347–480.

[B63] Kohlberg L. (1976). “Moral stages and moralization: The cognitive-developmental approach,” in Moral Development and Behavior: Theory and Research and Social Issues, ed T. Lickona (Holt, Rinehart, and Winston), 31–53.

[B64] Kohler U. Karlson K. B. Holm A. (2011). Comparing coefficients of nested nonlinear probability models. Stata J. 11, 420–438. 10.1177/1536867X1101100306

[B65] Koopmans R. (2013). Multiculturalism and immigration: a contested field in cross-national comparison. Ann. Rev. Sociol. 39, 147–169. 10.1146/annurev-soc-071312-145630

[B66] Kraaykamp G. Cemalcilar Z. Tosun J. (2019). Transmission of work attitudes and values: comparisons, consequences, and implications. Ann. Am. Acad Polit. Soc. Sci. 682, 8–24. 10.1177/0002716219831947

[B67] Kristen C. Dollmann J. (2009). Secondary effects of ethnic origin: students from turkish families at the transition to secondary education. Zeitschrift für Erziehungswissenschaft 12, 205–229.

[B68] Krueger S. Ebner C. Rohrbach-Schmidt D. (2022). Gender composition and the symbolic value of occupations: new evidence of a u-shaped relationship between gender and occupational prestige based on german microdata. Work Employ. Soc. 10.1177/09500170221117415

[B69] Law H. Schober P. (2021). Gendered occupational aspirations among German youth: Role of parental occupations, gender division of labour, and family structure. J. Family Res. 34, 1–26. 10.20377/jfr-603

[B70] Lawson K. M. Lee B. Crouter A. C. McHale S. M. (2018). Correlates of gendered vocational development from middle childhood to young adulthood. J. Vocat. Behav. 107, 209–221. 10.1016/j.jvb.2018.05.002

[B71] Leuze K. Strauß S. (2016). Why do occupations dominated by women pay less? How ‘female-typical' work tasks and working-time arrangements affect the gender wage gap among higher education graduates. Work Employ. Soc. 30, 802–820. 10.1177/0950017015624402

[B72] Levanon A. Grusky D. B. (2016). The Persistence of Extreme Gender Segregation in the Twenty-first Century. Am. J. Sociol. 122, 573–619. 10.1086/688628

[B73] Liben L. S. Bigler R. S. Krogh H. R. (2001). Pink and blue collar jobs: children's judgments of job status and job aspirations in relation to sex of worker. J. Exper. Child Psychol. 79, 346–363. 10.1006/jecp.2000.2611 11511128

[B74] Lloyd C. Mason G. Mayhew K. (2008). Low-Wage Work in the United Kingdom. New York: Russell Sage Foundation.

[B75] Logan J. R. Shin H.-j. (2012). Assimilation by the third generation? Marital choices of white ethnics at the dawn of the twentieth century. Soc. Sci. Res. 41, 1116–1125. 10.1016/j.ssresearch.2012.01.010 23017921 PMC3807942

[B76] Magnusson C. (2009). Gender, occupational prestige, and wages: a test of devaluation theory. Eur. Sociol. Rev. 25, 87–101. 10.1093/esr/jcn035

[B77] Magnusson C. (2013). More women, lower pay? Occupational sex composition, wages and wage growth. Acta Sociol. 56, 227–245. 10.1177/0001699313484480

[B78] Magnusson C. (2021). Flexible time – but is the time owned? Family friendly and family unfriendly work arrangements, occupational gender composition and wages: a test of the mother-friendly job hypothesis in Sweden, Community, Work and Family. Commun. Work Family 24, 291–314. 10.1080/13668803.2019.1697644

[B79] Mann A. DiPrete T. A. (2013). Trends in gender segregation in the choice of science and engineering majors. Soc. Sci. Res. 42, 1519–1541. 10.1016/j.ssresearch.2013.07.002 24090849 PMC3791309

[B80] Marini M. M. Fan P.-L. Finley E. Beutel A. M. (1996). Gender and job values. Sociol. Educ. 69, 49–65. 10.2307/2112723

[B81] Morgan S. L. Dafna G. Weeden K. A. (2013). Feeding the pipeline: Gender, occupational plans, and college major selection. Soc. Sci. Res. 42, 989–1005. 10.1016/j.ssresearch.2013.03.008 23721669

[B82] Möser S. (2022). Naïve or persistent optimism? The changing vocational aspirations of children of immigrants at the transition from school to work. Swiss J. Sociol. 48, 255–284. 10.2478/sjs-2022-0015

[B83] Moulton V. Flouri E. Joshi H. Sullivan A. (2018). Individual-level predictors of young children's aspirations. Res. Papers Educ. 33, 24–41. 10.1080/02671522.2016.122579737315095

[B84] Nauck B. (2023). Is ethnic retention a result of unmet educational aspirations? Academic career and ethnic identity of migrant minority youth in england, germany, the netherlands, and sweden. J. Int. Migr. Integr. 24, 261–280. 10.1007/s12134-020-00760-7

[B85] Nauck B. Lotter V. (2015). Parenting styles and perceived instrumentality of schooling in native, Turkish, and Vietnamese families in Germany. Zeitschrift für Erziehungswissenschaft 18, 845–869. 10.1007/s11618-015-0630-x

[B86] Nauck B. Schnoor B. (2015). Against all odds? Bildungserfolg in vietnamesischen und türkischen Familien in Deutschland. Kölner Zeitschrift für Soziol. Sozialpsychol. 67, 633–657. 10.1007/s11577-015-0345-2

[B87] Nitsche N. Grunow D. (2016). Housework over the course of the relationships: Gender ideology, resources, and the division of housework from a growth curve perspective. Adv. Life Course Res. 29, 80–94. 10.1016/j.alcr.2016.02.001

[B88] Ochsenfeld F. (2014). Why do women's fields of study pay less? A test of devaluation, human capital, and gender role theory. Eur. Sociol. Rev. 30, 536–548. 10.1093/esr/jcu060

[B89] OECD (2011). “Hours Worked, Self-Employment and Joblessness as Ingredients of Earnings Inequality,” in Divided We Stand: Why Inequality Keeps Rising (Paris: OECD Publishing) 167–191. 10.1787/9789264119536-8-en

[B90] OECD (2018). The Resilience of Students with an Immigrant Background: Factors that Shape Well-being, OECD Reviews of Migrant Education. Paris: OECD Publishing. 10.1787/9789264292093-en

[B91] OECD (2023). Wage levels (indicator). 10.1787/0a1c27bc-en

[B92] Office for National Statistics (2011). nomis: official census and labour market statistics. Annual Population Survey - Occupation by sex, employment status and full/part-time. Available online at: www.nomisweb.co.uk/datasets/aps210/reports/employment-by-status-and-occupation?compare=E92000001 (accessed May 15, 2023).

[B93] Official Statistics of Sweden. (2014). Employees 16–64 Years by Year, Occupation (SSYK 2012) and Sex. Available online at: https://www.statistikdatabasen.scb.se/pxweb/en/ssd/START__AM__AM0208__AM0208E/YREG54/ (accessed June 14, 2023).

[B94] Okamoto D. England P. (1999). Is there a supply side to occupational sex segregation? Sociol. Perspect. 42, 557–582. 10.2307/1389574

[B95] Platt L. Parsons S. (2017). Is the future female? Educational and occupational aspirations of teenage boys and girls in the UK. CLS working paper 2017/7.

[B96] Plenty S. M. Jonsson J. O. (2021). Students' occupational aspirations: can family relationships account for differences between immigrant and socioeconomic groups? Child Dev. 92, 157–173. 10.1111/cdev.13378 32573781 PMC7891578

[B97] Polavieja J. G. Fernández-Reino M. Ramos M. (2018). Are migrants selected on motivational orientations? Selectivity patterns amongst international migrants in Europe. Eur. Sociol. Rev. 34, 570–588. 10.1093/esr/jcy025

[B98] Polavieja J. G. Platt L. (2014). Nurse or mechanic? The role of parental socialization and children's personality in the formation of sex-typed occupational aspirations. Soc. Forces 93, 31–61. 10.1093/sf/sou051

[B99] Prix I. Kilpi-Jakonen E. (2022). Not in a class of one's own: social origin differentials in applying to gender-(a)typical fields of study across the educational hierarchy. Eur. Sociol. Rev. 38, 920–941. 10.1093/esr/jcac007

[B100] Pryor R. G. L. (1983). Sex differences in the levels of generality of values/preferences related to work. J. Vocat. Behav. 23, 233–241. 10.1016/0001-8791(83)90037-4

[B101] Reskin B. (1993). Sex segregation in the workplace. Ann. Rev. Sociol. 19, 241–270. 10.1146/annurev.so.19.080193.001325

[B102] Ridgeway C. L. Correll S. J. (2004). Unpacking the gender system:a theoretical perspective on gender beliefs and social relations. Gender Soc. 18, 510–531. 10.1177/0891243204265269

[B103] Risman B. J. (2004). Gender as a social structure:theory wrestling with activism. Gender Soc. 18, 429–450. 10.1177/0891243204265349

[B104] Risman B. J. (2017). 2016 southern sociological society presidential address: are millennials cracking the gender structure? Soc. Curr. 4, 208–227. 10.1177/2329496517697145

[B105] Röder A. (2014). Explaining religious differences in immigrants' gender role attitudes: the changing impact of origin country and individual religiosity. Ethnic Rac. Stud. Higher Educ. 37, 2615–2635. 10.1080/01419870.2013.854919

[B106] Röder A. Mühlau P. (2014). Are they acculturating? europe's immigrants and gender egalitarianism. Soc. Forces 92, 899–928. 10.1093/sf/sot126

[B107] Ros M. Schwartz S. H. Surkiss S. (1999). Basic individual values, work values, and the meaning of work. Appl. Psychol. 48, 49–71. 10.1111/j.1464-0597.1999.tb00048.x22774866

[B108] Sánchez Guerrero L. Schober P. S. (2021). Socialisation influences on gender ideologies of immigrant and native youth in Germany, England, Sweden and the Netherlands. Sex Roles 85, 113–127. 10.1007/s11199-020-01208-z 33311836 PMC7719056

[B109] Schieckoff B. Diehl C. (2021). The labor market participation of recently-arrived immigrant women in Germany. J. Family Res. 33, 322–350. 10.20377/jfr-462

[B110] Schober P. S. (2013). The parenthood effect on gender inequality: Explaining the change in paid and domestic work when British couples become parents. Eur. Sociol. Rev. 29, 74–85. 10.1093/esr/jcr041

[B111] Schoon I. (2001). Teenage job aspirations and career attainment in adulthood: A 17-year follow-up study of teenagers who aspired to become scientists, health professionals, or engineers. Int. J. Behav. Develop. 25, 124–132. 10.1080/01650250042000186

[B112] Schoon I. Eccles J. S. (2014). “Conceptualizing gender differences in aspirations and attainment: A life course perspective,” in Gender Differences in Aspirations and Attainment: A Life Course Perspective, eds. I. Schoon and J. S. Eccles (London: Cambridge University Press) 3–26. 10.1017/CBO9781139128933

[B113] Schoon I. Parsons S. (2002). Teenage aspirations for future careers and occupational outcomes. J. Vocat. Behav. 60, 262–288. 10.1006/jvbe.2001.1867

[B114] Selman R. L. (1980). The Growth of Interpersonal Understanding: Developmental and Clinical Analyses. New York, NY: Academic Press.

[B115] Sigle-Rushton W. Waldfogel J. (2007). Motherhood and women's earnings in Anglo-American, Continental European, and Nordic Countries. Feminist Econ. 13, 55–91. 10.1080/13545700601184849

[B116] Sikora J. Saha L. J. (2009). Gender and professional career plans of high school students in comparative perspective. Educ. Res. Evalut. 15, 385–403. 10.1080/13803610903087060

[B117] Stahl J. F. Schober P. S. (2018). Convergence or Divergence? Educational Discrepancies in Work-Care Arrangements of Mothers with Young Children in Germany. Work, Employ. Soc. 32, 629–649. 10.1177/0950017017692503

[B118] Statistisches Bundesamt. (2014). Frauenanteile_KldB5_Zensus2011.csv (Data available upon request to the Statistisches Bundesamt, Wiesbaden).

[B119] Stoet G. Geary D. C. (2022). Sex differences in adolescents' occupational aspirations: Variations across time and place. PLoS ONE 17, e0261438. 10.1371/journal.pone.0261438 35081124 PMC8791526

[B120] Strauss H. Maisonneuve C. (2009). The Wage Premium on Tertiary Education: New Estimates for 21 OECD Countries in OECD Journal: Economic Studies. 1, 1–29. 10.1787/eco_studies-v2009-art7-en

[B121] Taylor C. J. (2010). Occupational sex composition and the gendered availability of workplace support. Gender Soc. 24, 189–212. 10.1177/0891243209359912

[B122] Teig S. Susskind J. E. (2008). Truck driver or nurse? The impact of gender roles and occupational status on children's occupational preferences. Sex Roles 58, 848–863. 10.1007/s11199-008-9410-x

[B123] van De Werfhorst H. G. Van Tubergen F. (2007). Ethnicity, schooling, and merit in the Netherlands. Ethnicities 7, 416–444. 10.1177/1468796807080236

[B124] van der Vleuten M. Jaspers E. Maas I. van der Lippe T. (2016). Boys' and girls' educational choices in secondary education: The role of gender ideology. Educ. Stud. 42, 181–200. 10.1080/03055698.2016.1160821

[B125] Weisgram E. S. Bigler R. S. Liben L. S. (2010). Gender, values, and occupational interests among children, adolescents, and adults. Child Development. 81, 778–796. 10.1111/j.1467-8624.2010.01433.x 20573104

[B126] Weisgram E. S. Dinella L. M. Fulcher M. (2011). The role of masculinity/femininity, values, and occupational value affordances in shaping young men's and women's occupational choices. Sex Roles 65, 243–258. 10.1007/s11199-011-9998-0

[B127] Wicht A. Siembab M. (2022). Ethnic differences in gender-typical occupational orientations among adolescents in Germany. Soc. Inclus. 10, 5092. 10.17645/si.v10i2.5092

