# Supplementary material for: Gender typicality of occupational aspirations among immigrant and native youth: the role of gender ideology, educational aspirations, and work values
Source: Front Sociol. 2023 Jun 27;8:1161131. doi: 10.3389/fsoc.2023.1161131 (PMC10335811; doi:10.3389/fsoc.2023.1161131)
Supplement: Supplementary file 1 [file Data_Sheet_1.docx]

Gender Typicality of Occupational Aspirations Among Immigrant and Native Youth: The Role of Gender Ideology, Educational Aspirations, and Work Values

Ludovica Gambaro, Janna Wilhelm, Pia Schober

**Supplemental Material**

*Figure S1:* Predictive Margins of Reduced Multinomial Logistic Regression Model of Occupational Aspirations by Gender and Immigrant Background Group – England Sample

Note: Control variables: parental education, country.

| *Table S1:* Full Multinomial Regression Model with Subgroup Interactions for Gender Ideologies and Educational Aspirations (Weighted Sample). | | | | | | | | | | | | | |  |  |  |  |
| --- | --- | --- | --- | --- | --- | --- | --- | --- | --- | --- | --- | --- | --- | --- | --- | --- | --- |
|  | | Model 1: Occupational Aspirations | | | | | | Model 2: Occupational Aspirations | | | | | |  |  |  |  |
|  |  | Masculine | | Feminine | | Ultra-Feminine | | Masculine | | Feminine | | Ultra-Feminine | |  |  |  |  |
| Immigrant-origin boys ^a^ | | -0.33 | | -0.05 | | 0.21 | | -1.24* | | -1.00 | | -0.55 | |  |  |  |  |
|  | | (0.20) | | (0.19) | | (0.32) | | (0.61) | | (0.70) | | (0.91) | |  |  |  |  |
| Non-immigrant girls ^a^ | | -1.80*** | | 0.44*** | | 2.35*** | | -2.27*** | | 0.74 | | 3.12*** | |  |  |  |  |
|  | | (0.17) | | (0.13) | | (0.20) | | (0.40) | | (0.43) | | (0.45) | |  |  |  |  |
| Immigrant-origin girls ^a^ | | -2.18*** | | 0.43** | | 1.73*** | | -2.16** | | 1.31* | | 3.18*** | |  |  |  |  |
|  | | (0.20) | | (0.14) | | (0.22) | | (0.73) | | (0.54) | | (0.59) | |  |  |  |  |
| Gender ideologies (GI) | | -0.01 | | 0.04 | | -0.03 | | -0.02 | | -0.01 | | -0.25** | |  |  |  |  |
|  | | (0.07) | | (0.10) | | (0.16) | | (0.06) | | (0.06) | | (0.08) | |  |  |  |  |
| Immigrant-origin boys x GI | | -0.10 | | -0.04 | | 0.16 | | - | | - | | - | |  |  |  |  |
|  | | (0.18) | | (0.18) | | (0.30) | | - | | - | | - | |  |  |  |  |
| Non-immigrant girls x GI | | 0.09 | | -0.09 | | -0.23 | | - | | - | | - | |  |  |  |  |
|  | | (0.21) | | (0.14) | | (0.20) | | - | | - | | - | |  |  |  |  |
| Immigrant-origin girls x GI | | -0.05 | | -0.16 | | -0.52* | | - | | - | | - | |  |  |  |  |
|  | | (0.20) | | (0.14) | | (0.21) | |  | |  | |  | |  |  |  |  |
| EA: Secondary school degree (EA1) ^b^ | | -0.52* | | -0.20 | | -0.82*** | | -0.44 | | 0.23 | | -1.36** | |  |  |  |  |
|  | | (0.21) | | (0.23) | | (0.23) | | (0.29) | | (0.39) | | (0.50) | |  |  |  |  |
| EA: University degree (EA2) ^b^ | | -1.40*** | | -0.21 | | -1.56*** | | -1.65*** | | -0.15 | | -0.37 | |  |  |  |  |
|  | | (0.21) | | (0.22) | | (0.23) | | (0.27) | | (0.35) | | (0.43) | |  |  |  |  |
| Immigrant-origin boys x EA1 | | - | | - | | - | | 0.56 | | 0.56 | | 1.08 | |  |  |  |  |
|  | | - | | - | | - | | (0.81) | | (0.87) | | (1.15) | |  |  |  |  |
| Non-immigrant girls x EA1 | | - | | - | | - | | -0.00 | | -0.75 | | 0.36 | |  |  |  |  |
|  | | - | | - | | - | | (0.49) | | (0.52) | | (0.59) | |  |  |  |  |
| Immigrant-origin girls x EA1 | | - | | - | | - | | -1.63 | | -1.62* | | 0.10 | |  |  |  |  |
|  | | - | | - | | - | | (0.85) | | (0.65) | | (0.74) | |  |  |  |  |
| Immigrant-origin boys x EA2 | | - | | - | | - | | 1.19 | | 1.13 | | 0.46 | |  |  |  |  |
|  | | - | | - | | - | | (0.63) | | (0.72) | | (0.97) | |  |  |  |  |
| Non-immigrant girls x EA2 | | - | | - | | - | | 0.83 | | -0.21 | | -1.33** | |  |  |  |  |
|  | | - | | - | | - | | (0.44) | | (0.45) | | (0.51) | |  |  |  |  |
| Immigrant-origin girls x EA2 | | - | | - | | - | | 0.37 | | -0.78 | | -2.22*** | |  |  |  |  |
|  | | - | | - | | - | | (0.77) | | (0.56) | | (0.64) | |  |  |  |  |
| WV: High income | | -0.11* | | -0.16** | | -0.22** | | -0.11* | | -0.16** | | -0.22** | |  |  |  |  |
|  | | (0.05) | | (0.05) | | (0.07) | | (0.05) | | (0.05) | | (0.07) | |  |  |  |  |
| WV: Help others | | -0.00 | | 0.26*** | | 0.68*** | | -0.01 | | 0.25*** | | 0.70*** | |  |  |  |  |
|  | | (0.06) | | (0.05) | | (0.08) | | (0.06) | | (0.05) | | (0.08) | |  |  |  |  |
| WV: Think and solve problems | | 0.11 | | -0.08 | | -0.19** | | 0.11 | | -0.08 | | -0.19** | |  |  |  |  |
|  | | (0.06) | | (0.05) | | (0.07) | | (0.06) | | (0.05) | | (0.07) | |  |  |  |  |
| […] | |  | |  | |  | |  | |  | |  | |  |  |  |  |
| *N* | | 8,319 | | 8,319 | | 8,319 | | 8,319 | | 8,319 | | 8,319 | |  |  |  |  |
| Note: GI = gender ideologies. EA = educational aspirations. WV = work values. Robust standard errors in parentheses. ^a^ Ref.: Non-immigrant boys. ^b^ Ref.: Below secondary school degree or no degree. Control variables not shown. Ref.: Integrated. * p<0.05, ** p<0.01, *** p<0.001 | | | | | | | | | | | | | |  |  |  |  |
| Table S2: Full Multinomial Regression Model with Subgroup Interactions for Gender Ideologies and Educational Aspirations (Weighted Sample). | | | | | | | | | | | | | | | | | |
|  | Model 3: Occupational Aspirations | | | | | | Model 4: Occupational Aspirations | | | | | | Model 5: Occupational Aspirations | | | |  |
|  | Masculine | | Feminine | | Ultra-Feminine | | Masculine | | Feminine | | Ultra-Feminine | | Masculine | | Feminine | Ultra-Feminine |  |
| Immigrant-origin boys ^a^ | -0.25 | | -0.01 | | -0.15 | | -0.32 | | -0.02 | | 0.22 | | -0.29 | | -0.04 | 0.24 |  |
|  | (0.18) | | (0.20) | | (0.34) | | (0.18) | | (0.19) | | (0.31) | | (0.18) | | (0.19) | (0.31) |  |
| Non-immigrant girls ^a^ | -1.76*** | | 0.44*** | | 2.43*** | | -1.73*** | | 0.49*** | | 2.44*** | | -1.74*** | | 0.45*** | 2.53*** |  |
|  | (0.15) | | (0.12) | | (0.21) | | (0.16) | | (0.12) | | (0.21) | | (0.15) | | (0.12) | (0.21) |  |
| Immigrant-origin girls ^a^ | -2.17*** | | 0.47*** | | 1.86*** | | -2.18*** | | 0.36* | | 1.99*** | | -2.18*** | | 0.44** | 1.91*** |  |
|  | (0.20) | | (0.14) | | (0.23) | | (0.21) | | (0.14) | | (0.23) | | (0.21) | | (0.14) | (0.23) |  |
| Gender ideologies (GI) | -0.03 | | -0.01 | | -0.25** | | -0.03 | | -0.01 | | -0.25** | | -0.03 | | -0.02 | -0.26** |  |
|  | (0.06) | | (0.06) | | (0.08) | | (0.06) | | (0.06) | | (0.08) | | (0.06) | | (0.06) | (0.08) |  |
| EA: Secondary school degree (EA1)^b^ | -0.53* | | -0.21 | | -0.83*** | | -0.51* | | -0.18 | | -0.80*** | | -0.53* | | -0.20 | -0.80*** |  |
|  | (0.21) | | (0.23) | | (0.23) | | (0.21) | | (0.23) | | (0.23) | | (0.21) | | (0.23) | (0.23) |  |
| EA: University degree (EA2) ^b^ | -1.41*** | | -0.23 | | -1.58*** | | -1.40*** | | -0.21 | | -1.57*** | | -1.40*** | | -0.22 | -1.56*** |  |
|  | (0.21) | | (0.22) | | (0.23) | | (0.21) | | (0.22) | | (0.23) | | (0.21) | | (0.22) | (0.23) |  |
| WV: High income (WV1) | -0.04 | | -0.04 | | -0.05 | | -0.11* | | -0.16** | | -0.22** | | -0.11* | | -0.16** | -0.21** |  |
|  | (0.08) | | (0.11) | | (0.17) | | (0.06) | | (0.05) | | (0.07) | | (0.05) | | (0.05) | (0.07) |  |
| Immigrant-origin boys x WV1 | -0.19 | | -0.19 | | 0.42 | |  | |  | |  | |  | |  |  |  |
|  | (0.17) | | (0.19) | | (0.34) | |  | |  | |  | |  | |  |  |  |
| Non-immigrant girls x WV1 | -0.09 | | -0.13 | | -0.18 | |  | |  | |  | |  | |  |  |  |
|  | (0.12) | | (0.13) | | (0.20) | |  | |  | |  | |  | |  |  |  |
| Immigrant-origin girls x WV1 | -0.09 | | -0.36* | | -0.40 | |  | |  | |  | |  | |  |  |  |
|  | (0.19) | | (0.14) | | (0.22) | |  | |  | |  | |  | |  |  |  |
| WV: Help others (WV2) | -0.00 | | 0.26*** | | 0.68*** | | -0.05 | | 0.12 | | 0.40* | | -0.00 | | 0.26*** | 0.67*** |  |
|  | (0.06) | | (0.05) | | (0.08) | | (0.07) | | (0.08) | | (0.20) | | (0.06) | | (0.05) | (0.08) |  |
| Immigrant-origin boys x WV2 |  | |  | |  | | -0.20 | | -0.04 | | -0.09 | |  | |  |  |  |
|  |  | |  | |  | | (0.12) | | (0.14) | | (0.34) | |  | |  |  |  |
| Non-immigrant girls x WV2 |  | |  | |  | | 0.16 | | 0.21* | | 0.45* | |  | |  |  |  |
|  |  | |  | |  | | (0.16) | | (0.11) | | (0.22) | |  | |  |  |  |
| Immigrant-origin girls x WV2 |  | |  | |  | | 0.09 | | 0.38** | | 0.00 | |  | |  |  |  |
|  |  | |  | |  | | (0.21) | | (0.14) | | (0.23) | |  | |  |  |  |
| WV: Think/solve problems (WV3) | 0.11 | | -0.08 | | -0.19** | | 0.11 | | -0.08 | | -0.18** | | 0.08 | | -0.12 | -0.44** |  |
|  | (0.06) | | (0.05) | | (0.07) | | (0.06) | | (0.05) | | (0.07) | | (0.08) | | (0.08) | (0.17) |  |
| Immigrant-origin boys x WV3 |  | |  | |  | |  | |  | |  | | -0.02 | | 0.07 | 0.16 |  |
|  |  | |  | |  | |  | |  | |  | | (0.14) | | (0.15) | (0.34) |  |
| Non-immigrant girls x WV3 |  | |  | |  | |  | |  | |  | | 0.10 | | 0.10 | 0.33 |  |
|  |  | |  | |  | |  | |  | |  | | (0.12) | | (0.11) | (0.18) |  |
| Immigrant-origin girls x WV3 |  | |  | |  | |  | |  | |  | | -0.03 | | -0.00 | 0.21 |  |
|  |  | |  | |  | |  | |  | |  | | (0.21) | | (0.13) | (0.21) |  |
| […] |  | |  | |  | |  | |  | |  | |  | |  |  |  |
| *N* | 8,319 | | 8,319 | | 8,319 | | 8,319 | | 8,319 | | 8,319 | | 8,319 | | 8,319 | 8,319 |  |
| Note: GI = gender ideologies. EA = educational aspirations. WV = work values. Robust standard errors in parentheses. ^a^ Ref.: Non-immigrant boys. ^b^ Ref.: Below secondary school degree or no degree. Control variables not shown. Ref.: Integrated. * p<0.05, ** p<0.01, *** p<0.001 | | | | | | | | | | | | | | | | |  |

| *Table S3:* Descriptive Statistics by Subgroup – England (Weighted Sample). | | | | | | | |  |
| --- | --- | --- | --- | --- | --- | --- | --- | --- |
|  | Mean (SD) / Proportion (n) | | | | | | | Sig. ^a^ |
|  |  | | Boys | | | Girls | |  |
|  | Full sample | NB | | IB | NB | | IB |  |
| OA: Masculine | .25 (447) | .47 (255) | | .41 (122) | .10 (57) | | .05 (15) | b |
| OA: Integrated | .32 (563) | .29 (159) | | .35 (101) | .29 (172) | | .48 (161) | b |
| OA: Feminine | .32 (561) | .22 (121) | | .21 (62) | .40 (235) | | .40 (133) | ns |
| OA: Ultra-feminine | .12 (191) | .01 (8) | | .02 (7) | .22 (127) | | .07 (24) | b |
|  |  |  | |  |  | |  |  |
| Gender ideologies (0–1) | .76 (.36) | .65 (.41) | | .61 (.38) | .86 (.32) | | .79 (.34) | a, b |
|  |  |  | |  |  | |  |  |
| EA: < Upper sec. school degree | .13 (226) | .18 (99) | | .07 (21) | .13 (74) | | .02 (7) | a, b |
| EA: Upper sec. school degree | .15 (271) | .21 (113) | | .08 (24) | .14 (82) | | .10 (34) | a |
| EA: University degree | .72 (1265) | .61 (331) | | .85 (248) | .74 (436) | | .88 (294) | a, b |
|  |  |  | |  |  | |  |  |
| WV: High income (1–4) | 3.16 (.69) | 3.29 (.56) | | 3.46 (.60) | 3.23 (.61) | | 3.48 (.64) | a, b |
| WV: Help others (1–4) | 3.20 (.75) | 3.02 (.75) | | 2.99 (.91) | 3.33 (.68) | | 3.42 (.72) | ns |
| WV: Think/solve problems (1–4) | 3.16 (.69) | 3.12 (.71) | | 3.26 (.75) | 3.11 (.65) | | 3.35 (.70) | b |
|  |  |  | |  |  | |  |  |
| Parents without tertiary education | .60 (1055) | .66 (357) | | .59 (173) | .59 (347) | | .47 (158) | b |
| Parents with tertiary education | .40 (707) | .34 (186) | | .41 (120) | .41 (245) | | .53 (176) | b |
|  |  |  | |  |  | |  |  |
| *N* | 1762 | 634 | | 160 | 760 | | 208 | - |
| Note. NB = non-immigrant background. IB = immigrant background. OA = occupational aspirations. EA = educational aspirations. WV = work values. Due to the weighting, the number of participants does not add up to the overall number. Proportional values are followed by the number of adolescents in parentheses. Mean values are followed by their standard deviation. ^a^ Significance (p < 0.05) of the difference between non-immigrant and immigrant-origin boys (a) and girls (b; ns = not significant). | | | | | | | | |

| *Table S4:* Descriptive Statistics by Subgroup – Germany (Weighted Sample). | | | | | | | |  |
| --- | --- | --- | --- | --- | --- | --- | --- | --- |
|  | Mean (SD) / Proportion (n) | | | | | | | Sig. ^a^ |
|  |  | | Boys | | | Girls | |  |
|  | Full sample | NB | | IB | NB | | IB |  |
| OA: Masculine | .33 (768) | .60 (424) | | .49 (201) | .09 (68) | | .12 (50) | a |
| OA: Integrated | .24 (564) | .21 (147) | | .27 (114) | .26 (202) | | .27 (118) | ns |
| OA: Feminine | .28 (643) | .17 (119) | | .21 (88) | .37 (282) | | .40 (172) | ns |
| OA: Ultra-feminine | .15 (353) | .02 (17) | | .03 (12) | .29 (220) | | .22 (94) | ns |
|  |  |  | |  |  | |  |  |
| Gender ideologies (0–1) | .73 (.36) | .63 (.38) | | .60 (.38) | .85 (.30) | | .75 (.32) | b |
|  |  |  | |  |  | |  |  |
| EA: < Upper sec. school degree | .23 (546) | .28 (195) | | .27 (113) | .20 (157) | | .17 (72) | ns |
| EA: Upper sec. school degree | .33 (764) | .34 (239) | | .26 (107) | .34 (259) | | .32 (139) | a |
| EA: University degree | .44 (1019) | .39 (274) | | .47 (195) | .46 (355) | | .52 (224) | ns |
|  |  |  | |  |  | |  |  |
| WV: High income (1–4) | 3.09 (.60) | 3.12 (.59) | | 3.42 (.60) | 2.96 (.57) | | 3.20 (.58) | a, b |
| WV: Help others (1–4) | 3.00 (.73) | 2.78 (.71) | | 3.00 (.71) | 3.12 (.72) | | 3.32 (.69) | a, b |
| WV: Think/solve problems (1–4) | 3.04 (.73) | 3.03 (.77) | | 3.12 (.73) | 3.01 (.70) | | 3.12 (.71) | ns |
|  |  |  | |  |  | |  |  |
| Parents without tertiary education | .75 (1738) | .75 (528) | | .85 (353) | .73 (566) | | .71 (307) | a |
| Parents with tertiary education | .25 (590) | .25 (179) | | .15 (61) | .27 (206) | | .29 (128) | a |
|  |  |  | |  |  | |  |  |
| *N* | 2329 | 932 | | 202 | 968 | | 227 |  |
| Note. NB = non-immigrant background. IB = immigrant background. OA = occupational aspirations. EA = educational aspirations. WV = work values. Due to the weighting, the number of participants does not add up to the overall number. Proportional values are followed by the number of adolescents in parentheses. Mean values are followed by their standard deviation. ^a^ Significance (p < 0.05) of the difference between non-immigrant and immigrant-origin boys (a) and girls (b; ns = not significant). | | | | | | | | |

| *Table S5:* Descriptive Statistics by Subgroup – Netherlands (Weighted Sample). | | | | | | | |  |
| --- | --- | --- | --- | --- | --- | --- | --- | --- |
|  | Mean (SD) / Proportion (n) | | | | | | | Sig. ^a^ |
|  |  | | Boys | | | Girls | |  |
|  | Full sample | NB | | IB | NB | | IB |  |
| OA: Masculine | .31 (595) | .56 (387) | | .38 (77) | .09 (69) | | .02 (6) | ns |
| OA: Integrated | .24 (458) | .25 (176) | | .47 (97 | .21 (164) | | .17 (40) | ns |
| OA: Feminine | .27 (509) | .18 (123) | | .13 (26) | .34 (263) | | .56 (129) | b |
| OA: Ultra-feminine | .18 (351) | .01 (10) | | .02 (4) | .37 (287) | | .24 (55) | ns |
|  |  |  | |  |  | |  |  |
| Gender ideologies (0–1) | .66 (39) | .56 (.39) | | .63 (.33) | .75 (.37) | | .72 (.38) | ns |
|  |  |  | |  |  | |  |  |
| EA: < Upper sec. school degree | .07 (125) | .07 (52) | | .02 (4) | .06 (50) | | .04 (9) | a |
| EA: Upper sec. school degree | .33 (626) | .42 (292) | | .34 (69) | .26 (205) | | .08 (20) | b |
| EA: University degree | .61 (1163) | .51 (354) | | .65 (132) | .67 (528) | | .87 (200) | b |
|  |  |  | |  |  | |  |  |
| WV: High income (1–4) | 3.08 (.55) | 3.14 (.54) | | 3.43 (.52) | 2.98 (.55) | | 3.10 (.56) | a |
| WV: Help others (1–4) | 3.12 (.65) | 2.93 (.62) | | 3.08 (.52) | 3.28 (.64) | | 3.49 (.56) | a, b |
| WV: Think/solve problems (1–4) | 3.10 (.58) | 3.12 (.61) | | 3.16 (.51) | 3.05 (.55) | | 3.35 (.58) | b |
|  |  |  | |  |  | |  |  |
| Parents without tertiary education | .58 (1116) | .60 (421) | | .65 (133) | .56 (435) | | .57 (130) | ns |
| Parents with tertiary education | .42 (798) | .40 (276) | | .35 (72) | .44 (347) | | .43 (99) | ns |
|  |  |  | |  |  | |  |  |
| *N* | 1914 | 861 | | 91 | 854 | | 100 |  |
| Note. NB = non-immigrant background. IB = immigrant background. OA = occupational aspirations. EA = educational aspirations. WV = work values. Due to the weighting, the number of participants does not add up to the overall number. Proportional values are followed by the number of adolescents in parentheses. Mean values are followed by their standard deviation. ^a^ Significance (p < 0.05) of the difference between non-immigrant and immigrant-origin boys (a) and girls (b; ns = not significant). | | | | | | | | |

| *Table S6:* Descriptive Statistics by Subgroup – Sweden (Weighted Sample). | | | | | | | | |  |
| --- | --- | --- | --- | --- | --- | --- | --- | --- | --- |
|  | | Mean (SD) / Proportion (n) | | | | | | | Sig. ^a^ |
|  | |  | | Boys | | | Girls | |  |
|  | Full sample | | NB | | IB | NB | | IB |  |
| OA: Masculine | .31 (711) | | .55 (388) | | .47 (181) | .10 (78) | | .07 (31) | ns |
| OA: Integrated | .28 (641) | | .22 (159) | | .20 (78) | .33 (252) | | .32 (148) | ns |
| OA: Feminine | .28 (655) | | .19 (132) | | .28 (108) | .34 (259) | | .41 (189) | a |
| OA: Ultra-feminine | .13 (307) | | .04 (28) | | .05 (18) | .22 (167) | | .21 (98) | ns |
|  |  | |  | |  |  | |  |  |
| Gender ideologies (0–1) | .85 (.35) | | .75 (.41) | | .64 (.39) | .99 (.20) | | .86 (.31) | a, b |
|  |  | |  | |  |  | |  |  |
| EA: < Upper sec. school degree | .01 (14) | | .01 (8) | | .00 (1) | .00 (1) | | .01 (3) | ns |
| EA: Upper sec. school degree | .16 (378) | | .25 (176) | | .12 (58) | .12 (89) | | .06 (28) | a, b |
| EA: University degree | .83 (1922) | | .74 (522) | | .87 (336) | .88 (666) | | .93 (435) | a, b |
|  |  | |  | |  |  | |  |  |
| WV: High income (1–4) | 3.21 (.63) | | 3.28 (.65) | | 3.40 (.62) | 3.08 (.59) | | 3.27 (.63) | a, b |
| WV: Help others (1–4) | 3.04 (.79) | | 2.81 (.79) | | 3.09 (.85) | 3.15 (.74) | | 3.42 (.68) | a, b |
| WV: Think/solve problems (1–4) | 3.22 (.71) | | 3.21 (.72) | | 3.32 (.72) | 3.14 (.69) | | 3.42 (.69) | b |
|  |  | |  | |  |  | |  |  |
| Parents without tertiary education | .46 (1070) | | .45 (321) | | .48 (186) | .45 (342) | | .51 (235) | ns |
| Parents with tertiary education | .54 (1243) | | .55 (386) | | .52 (199) | .55 (413) | | .49 (231) | ns |
|  |  | |  | |  |  | |  |  |
| *N* | 2314 | | 904 | | 217 | 928 | | 265 |  |
| Note. NB = non-immigrant background. IB = immigrant background. OA = occupational aspirations. EA = educational aspirations. WV = work values. Due to the weighting, the number of participants does not add up to the overall number. Proportional values are followed by the number of adolescents in parentheses. Mean values are followed by their standard deviation. ^a^ Significance (p < 0.05) of the difference between non-immigrant and immigrant-origin boys (a) and girls (b; ns = not significant). | | | | | | | | | |

|  |
| --- |

| *Table S7:* Predictive Margins of Occupational Aspirations by Subgroup Estimated After the Reduced Multinomial Logistic Regression Model Including Only Control Variables (Weighted Samples) | | | | |
| --- | --- | --- | --- | --- |
|  | Masculine | Integrated | Feminine | Ultra-Feminine |
| **England** |  |  |  |  |
| Non-immigrant boys ^a^ | 0.463 | 0.301 | 0.221 | 0.014 |
| Immigrant-origin boys ^a^ | 0.418 | 0.344 | 0.214 | 0.023 |
| Non-immigrant girls ^a^ | 0.096 | 0.289 | 0.398 | 0.228 |
| Immigrant-origin girls ^a^ | 0.047 | 0.464 | 0.409 | 0.080 |
| *N* | 1,762 | 1,762 | 1,762 | 1,762 |
| **Germany** |  |  |  |  |
| Non-immigrant boys ^a^ | 0.599 | 0.208 | 0.168 | 0.024 |
| Immigrant-origin boys ^a^ | 0.474 | 0.284 | 0.215 | 0.027 |
| Non-immigrant girls ^a^ | 0.088 | 0.260 | 0.364 | 0.287 |
| Immigrant-origin girls ^a^ | 0.117 | 0.267 | 0.395 | 0.222 |
| *N* | 2,329 | 2,329 | 2,329 | 2,329 |
| **Netherlands** |  |  |  |  |
| Non-immigrant boys ^a^ | 0.552 | 0.254 | 0.179 | 0.015 |
| Immigrant-origin boys ^a^ | 0.367 | 0.481 | 0.130 | 0.021 |
| Non-immigrant girls ^a^ | 0.089 | 0.208 | 0.332 | 0.371 |
| Immigrant-origin girls ^a^ | 0.025 | 0.173 | 0.561 | 0.241 |
| *N* | 1,914 | 1,914 | 1,914 | 1,914 |
| **Sweden** |  |  |  |  |
| Non-immigrant boys ^a^ | 0.550 | 0.224 | 0.186 | 0.039 |
| Immigrant-origin boys ^a^ | 0.468 | 0.203 | 0.282 | 0.047 |
| Non-immigrant girls ^a^ | 0.103 | 0.333 | 0.342 | 0.222 |
| Immigrant-origin girls ^a^ | 0.066 | 0.320 | 0.408 | 0.207 |
| *N* | 2,314 | 2,314 | 2,314 | 2,314 |
| Note: Control variables: parental education. | | | | |

| *Table S8:* Average Marginal Effects of Full Multinomial Logistic Regression Model of Occupational Aspirations (Weighted Sample) – England | | | | |
| --- | --- | --- | --- | --- |
|  | Masculine | Integrated | Feminine | Ultra-Feminine |
| Immigrant-origin boys ^a^ | 0.009 | 0.008 | -0.031 | 0.015 |
| Non-immigrant girls ^a^ | -0.318*** | -0.022 | 0.137*** | 0.203*** |
| Immigrant-origin girls ^a^ | -0.368*** | 0.131* | 0.135** | 0.102** |
|  |  |  |  |  |
| Gender ideologies | -0.005 | 0.024 | -0.006 | -0.012 |
|  |  |  |  |  |
| EA: Secondary school degree ^b^ | 0.005 | 0.156** | -0.005 | -0.156** |
| EA: University degree ^b^ | -0.196*** | 0.225*** | 0.184*** | -0.213*** |
|  |  |  |  |  |
| WV: High income | 0.009 | 0.023 | -0.024 | -0.007 |
| WV: Help others | -0.027 | -0.046** | 0.053** | 0.020* |
| WV: Think and solve problems | 0.029* | 0.005 | -0.024 | -0.010 |
|  |  |  |  |  |
| Parents with tertiary education | -0.016 | 0.109** | -0.064* | -0.029 |
|  |  |  |  |  |
| *N* | 1,762 | 1,762 | 1,762 | 1,762 |
| Note: EA = educational aspirations. WV = work values. ^a^ Ref.: Non-immigrant boys. ^b^ Ref.: Below secondary school degree or no degree. ^c^ Ref.: England. * p<0.05, ** p<0.01, *** p<0.001 | | | | |

| Table S9: Average Marginal Effects of Full Multinomial Logistic Regression Model of Occupational Aspirations (Weighted Sample) – Germany | | | | |
| --- | --- | --- | --- | --- |
|  | Masculine | Integrated | Feminine | Ultra-Feminine |
| Immigrant-origin boys ^a^ | -0.104* | 0.073 | 0.030 | 0.001 |
| Non-immigrant girls ^a^ | -0.488*** | 0.067* | 0.184*** | 0.237*** |
| Immigrant-origin girls ^a^ | -0.450*** | 0.086* | 0.201*** | 0.164*** |
|  |  |  |  |  |
| Gender ideologies | 0.010 | -0.000 | -0.009 | -0.002 |
|  |  |  |  |  |
| EA: Secondary school degree ^b^ | -0.092* | 0.073 | 0.057 | -0.039 |
| EA: University degree ^b^ | -0.140*** | 0.084* | 0.177*** | -0.121*** |
|  |  |  |  |  |
| WV: High income | -0.008 | 0.028* | -0.002 | -0.018 |
| WV: Help others | -0.037** | -0.041** | 0.007 | 0.071*** |
| WV: Think and solve problems | 0.030* | 0.008 | -0.027* | -0.011 |
|  |  |  |  |  |
| Academic school track | -0.022 | 0.071* | 0.006 | -0.054* |
| Parents with tertiary education | -0.014 | 0.051 | 0.007 | -0.044 |
|  |  |  |  |  |
| *N* | 2,329 | 2,329 | 2,329 | 2,329 |
| Note: EA = educational aspirations. WV = work values. ^a^ Ref.: Non-immigrant boys. ^b^ Ref.: Below secondary school degree or no degree. ^c^ Ref.: England. * p<0.05, ** p<0.01, *** p<0.001 | | | | |

| *Table S10:* Average Marginal Effects of Full Multinomial Logistic Regression Model of Occupational Aspirations (Weighted Sample) – Netherlands | | | | |
| --- | --- | --- | --- | --- |
|  | Masculine | Integrated | Feminine | Ultra-Feminine |
| Immigrant-origin boys ^a^ | -0.150 | 0.185 | -0.044 | 0.010 |
| Non-immigrant girls ^a^ | -0.441*** | -0.026 | 0.110* | 0.357*** |
| Immigrant-origin girls ^a^ | -0.504*** | -0.056 | 0.336*** | 0.224*** |
|  |  |  |  |  |
| Gender ideologies | -0.001 | 0.020 | 0.018 | -0.037* |
|  |  |  |  |  |
| EA: Secondary school degree ^b^ | -0.042 | 0.048 | 0.005 | -0.012 |
| EA: University degree ^b^ | -0.130 | 0.185*** | 0.004 | -0.059 |
|  |  |  |  |  |
| WV: High income | -0.011 | 0.046* | -0.028 | -0.007 |
| WV: Help others | 0.018 | -0.094*** | 0.010 | 0.066*** |
| WV: Think and solve problems | -0.007 | -0.012 | 0.011 | 0.008 |
|  |  |  |  |  |
| Academic school track | -0.110* | 0.024 | 0.160*** | -0.073* |
| Parents with tertiary education | -0.054 | 0.020 | 0.078 | -0.044 |
|  |  |  |  |  |
| *N* | 1,914 | 1,914 | 1,914 | 1,914 |
| Note: EA = educational aspirations. WV = work values. ^a^ Ref.: Non-immigrant boys. ^b^ Ref.: Below secondary school degree or no degree. ^c^ Ref.: England. * p<0.05, ** p<0.01, *** p<0.001 | | | | |

| *Table S11:* Average Marginal Effects of Full Multinomial Logistic Regression Model of Occupational Aspirations (Weighted Sample) – Sweden | | | | |
| --- | --- | --- | --- | --- |
|  | Masculine | Integrated | Feminine | Ultra-Feminine |
| Immigrant-origin boys ^a^ | -0.027 | -0.055 | 0.082* | 0.000 |
| Non-immigrant girls ^a^ | -0.385*** | 0.095** | 0.109*** | 0.181*** |
| Immigrant-origin girls ^a^ | -0.416*** | 0.067 | 0.188*** | 0.160*** |
|  |  |  |  |  |
| Gender ideologies | 0.002 | -0.009 | 0.040** | -0.033*** |
|  |  |  |  |  |
| EA: Secondary school degree ^b^ | 0.010 | 0.121* | 0.058 | -0.190 |
| EA: University degree ^b^ | -0.208 | 0.259*** | 0.159 | -0.210 |
|  |  |  |  |  |
| WV: High income | -0.001 | 0.013 | 0.002 | -0.014 |
| WV: Help others | -0.049*** | -0.004 | 0.006 | 0.047*** |
| WV: Think and solve problems | 0.040** | 0.012 | -0.010 | -0.042*** |
|  |  |  |  |  |
| Parents with tertiary education | -0.027 | 0.042 | 0.011 | -0.026 |
|  |  |  |  |  |
| *N* | 2,314 | 2,314 | 2,314 | 2,314 |
| Note: EA = educational aspirations. WV = work values. ^a^ Ref.: Non-immigrant boys. ^b^ Ref.: Below secondary school degree or no degree. ^c^ Ref.: England. * p<0.05, ** p<0.01, *** p<0.001 | | | | |

| *Table S12:* Comparing Subgroup Coefficients Of The Reduced Multinomial Model Including Only Control Variables Versus Models That Additionally Include Gender Ideology, Educational Aspirations, And Work Values (Ref.: Non-Immigrant Boys) – England | | | |
| --- | --- | --- | --- |
|  | Masculine | Feminine | Ultra-feminine |
| **Immigrant-origin boys** |  |  |  |
| Reduced model | -0.045 | -0.006 | 0.009 |
|  | (-0.89) | (-0.15) | (0.88) |
| Incl. gender ideology | -0.053 | -0.005 | 0.008 |
|  | (-1.04) | (-0.12) | (0.83) |
| Incl. educational aspirations | **0.016** | -0.033 | **0.014** |
|  | **(0.32)** | (-0.77) | **(1.24)** |
| Incl. WV: high income | -0.047 | -0.001 | 0.010 |
|  | (-0.91) | (-0.03) | (0.94) |
| Incl. WV: helping others | -0.045 | -0.006 | 0.009 |
|  | (-0.90) | (-0.14) | (0.90) |
| Incl. WV: thinking/solving problems | -0.050 | -0.005 | 0.009 |
|  | (-0.94) | (-0.13) | (0.89) |
| Incl. all mediators | **0.010** | -0.033 | **0.013** |
|  | **(0.21)** | (-0.76) | **(1.21)** |
| **Non-immigrant girls** |  |  |  |
| Reduced model | -0.368*** | 0.176*** | 0.203*** |
|  | (-10.76) | (4.62) | (8.52) |
| Incl. gender ideology | -0.358*** | 0.171*** | 0.212*** |
|  | (-10.20) | (4.42) | (8.21) |
| Incl. educational aspirations | -0.338*** | 0.162*** | 0.207*** |
|  | (-10.76) | (4.33) | (9.41) |
| Incl. WV: high income | -0.367*** | 0.175*** | 0.200*** |
|  | (-10.75) | (4.58) | (8.38) |
| Incl. WV: helping others | -0.359*** | 0.158*** | 0.196*** |
|  | (-10.27) | (4.06) | (8.39) |
| Incl. WV: thinking/solving problems | -0.368*** | 0.177*** | 0.202*** |
|  | (-10.80) | (4.67) | (8.47) |
| Incl. all mediators | -0.333*** | 0.159*** | 0.214*** |
|  | (-10.21) | (4.20) | (8.84) |
| **Immigrant-origin girls** |  |  |  |
| Reduced model | -0.417*** | 0.188*** | 0.065** |
|  | (-12.89) | (3.66) | (2.74) |
| Incl. gender ideology | -0.409*** | 0.186*** | 0.067** |
|  | (-12.60) | (3.69) | (2.72) |
| Incl. educational aspirations | **-0.378***** | 0.154** | **0.102**** |
|  | **(-11.99)** | (3.05) | **(3.15)** |
| Incl. WV: high income | -0.418*** | 0.196*** | 0.069** |
|  | (-13.00) | (3.91) | (2.77) |
| Incl. WV: helping others | -0.410*** | **0.159**** | 0.061* |
|  | (-12.30) | **(3.19)** | (2.55) |
| Incl. WV: thinking/solving problems | -0.419*** | 0.187*** | 0.067** |
|  | (-12.99) | (3.63) | (2.74) |
| Incl. all mediators | **-0.374***** | 0.153** | **0.103**** |
|  | **(-11.77)** | (3.09) | **(3.24)** |
| *N* | 1,762 | 1,762 | 1,762 |
| Note: WV = work value. Ref.: Integrated occupations. T-statistics in parentheses. Control variables: parental education, country. Asterisks refer to significance of coefficient (* p<0.05, ** p<0.01, *** p<0.001). Bold lettering indicates a significant difference compared to the reduced model (p<0.05). | | | |

| *Table S13:* Comparing Subgroup Coefficients Of The Reduced Multinomial Model Including Only Control Variables Versus Models That Additionally Include Gender Ideology, Educational Aspirations, And Work Values (Ref.: Non-Immigrant Girls) – England | | | |
| --- | --- | --- | --- |
|  | Masculine | Feminine | Ultra-feminine |
| **Non-immigrant boys** |  |  |  |
| Reduced model | 0.368*** | -0.176*** | -0.203*** |
|  | (10.76) | (-4.62) | (-8.52) |
| Incl. gender ideology | 0.358*** | -0.171*** | -0.212*** |
|  | (10.20) | (-4.42) | (-8.21) |
| Incl. educational aspirations | 0.338*** | -0.162*** | -0.207*** |
|  | (10.76) | (-4.33) | (-9.41) |
| Incl. WV: high income | 0.367*** | -0.175*** | -0.200*** |
|  | (10.75) | (-4.58) | (-8.38) |
| Incl. WV: helping others | 0.359*** | -0.158*** | -0.196*** |
|  | (10.27) | (-4.06) | (-8.39) |
| Incl. WV: thinking/solving problems | 0.368*** | -0.177*** | -0.202*** |
|  | (10.80) | (-4.67) | (-8.47) |
| Incl. all mediators | 0.333*** | -0.159*** | -0.214*** |
|  | (10.21) | (-4.20) | (-8.84) |
| **Immigrant-origin boys** |  |  |  |
| Reduced model | 0.322*** | -0.182*** | -0.194*** |
|  | (7.01) | (-3.90) | (-7.82) |
| Incl. gender ideology | 0.305*** | -0.176*** | -0.203*** |
|  | (6.44) | (-3.58) | (-7.67) |
| Incl. educational aspirations | 0.354*** | -0.196*** | -0.193*** |
|  | (7.60) | (-4.40) | (-8.14) |
| Incl. WV: high income | 0.320*** | -0.176*** | -0.190*** |
|  | (6.81) | (-3.68) | (-7.59) |
| Incl. WV: helping others | 0.314*** | -0.165*** | -0.186*** |
|  | (7.06) | (-3.46) | (-7.57) |
| Incl. WV: thinking/solving problems | 0.318*** | -0.183*** | -0.192*** |
|  | (6.71) | (-3.90) | (-7.73) |
| Incl. all mediators | 0.344*** | -0.193*** | -0.201*** |
|  | (7.19) | (-4.11) | (-7.80) |
| **Immigrant-origin girls** |  |  |  |
| Reduced model | -0.049* | 0.012 | -0.137*** |
|  | (-2.19) | (0.23) | (-4.20) |
| Incl. gender ideology | -0.050* | 0.015 | -0.144*** |
|  | (-2.20) | (0.29) | (-4.44) |
| Incl. educational aspirations | **-0.039** | -0.008 | **-0.105**** |
|  | **(-1.56)** | (-0.17) | **(-2.80)** |
| Incl. WV: high income | -0.05^* | 0.021 | -0.131*** |
|  | (-2.22) | (0.39) | (-3.91) |
| Incl. WV: helping others | -0.050* | 0.001 | -0.135*** |
|  | (-2.19) | (0.02) | (-4.24) |
| Incl. WV: thinking/solving problems | -0.051* | 0.009 | -0.134*** |
|  | (-2.25) | (0.18) | (-4.04) |
| Incl. all mediators | -0.040 | -0.005 | -0.112** |
|  | (-1.56) | (-0.11) | (-3.07) |
| *N* | 1,762 | 1,762 | 1,762 |
| Note: WV = work value. Ref.: Integrated occupations. T-statistics in parentheses. Control variables: parental education, country. Asterisks refer to significance of coefficient (* p<0.05, ** p<0.01, *** p<0.001). Bold lettering indicates a significant difference compared to the reduced model (p<0.05). | | | |
|  | | | |

| *Table S14:* Comparing Subgroup Coefficients Of The Reduced Multinomial Model Including Only Control Variables Versus Models That Additionally Include Gender Ideology, Educational Aspirations, And Work Values (Ref.: Non-Immigrant Boys) – Germany | | | |
| --- | --- | --- | --- |
|  | Masculine | Feminine | Ultra-feminine |
| **Immigrant-origin boys** |  |  |  |
| Reduced model | -0.126* | 0.046 | 0.002 |
|  | (-2.43) | (1.12) | (0.24) |
| Incl. gender ideology | -0.125* | 0.046 | 0.002 |
|  | (-2.42) | (1.12) | (0.23) |
| Incl. educational aspirations | -0.108* | 0.036 | 0.005 |
|  | (-2.01) | (0.91) | (0.42) |
| Incl. WV: high income | -0.109* | 0.044 | **0.005** |
|  | (-2.10) | (1.06) | **(0.44)** |
| Incl. WV: helping others | -0.116* | 0.040 | -0.001 |
|  | (-2.22) | (0.96) | (-0.24) |
| Incl. WV: thinking/solving problems | -0.129* | 0.049 | 0.002 |
|  | (-2.50) | (1.18) | (0.24) |
| Incl. all mediators | -0.0912 | 0.031 | 0.002 |
|  | (-1.73) | (0.78) | (0.15) |
| **Non-immigrant girls** |  |  |  |
| Reduced model | -0.511*** | 0.196*** | 0.263*** |
|  | (-16.92) | (5.87) | (10.54) |
| Incl. gender ideology | -0.513*** | 0.196*** | 0.268*** |
|  | (-17.04) | (5.66) | (9.97) |
| Incl. educational aspirations | -0.500*** | 0.185*** | 0.273*** |
|  | (-16.98) | (5.54) | (11.25) |
| Incl. WV: high income | -0.514*** | 0.199*** | 0.254*** |
|  | (-17.12) | (5.97) | (10.29) |
| Incl. WV: helping others | -0.498*** | 0.196*** | **0.236***** |
|  | (-15.84) | (5.78) | **(9.71)** |
| Incl. WV: thinking/solving problems | -0.511*** | 0.196*** | 0.264*** |
|  | (-16.89) | (5.85) | (10.54) |
| Incl. all mediators | -0.485*** | 0.184*** | 0.236*** |
|  | (-15.38) | (5.26) | (9.16) |
| **Immigrant-origin girls** |  |  |  |
| Reduced model | -0.483*** | 0.227*** | 0.198*** |
|  | (-11.99) | (5.18) | (6.16) |
| Incl. gender ideology | -0.484*** | 0.227*** | 0.199*** |
|  | (-12.05) | (5.19) | (6.14) |
| Incl. educational aspirations | -0.469*** | 0.206*** | 0.217*** |
|  | (-11.61) | (4.79) | (6.80) |
| Incl. WV: high income | -0.483*** | 0.224*** | 0.205*** |
|  | (-11.95) | (5.10) | (6.22) |
| Incl. WV: helping others | -0.462*** | 0.227*** | **0.150***** |
|  | (-10.73) | (5.03) | **(4.77)** |
| Incl. WV: thinking/solving problems | -0.484*** | 0.229*** | 0.197*** |
|  | (-12.14) | (5.24) | (6.10) |
| Incl. all mediators | -0.446*** | 0.201*** | 0.174*** |
|  | (-10.14) | (4.54) | (5.41) |
| *N* | 2,329 | 2,329 | 2,329 |
| Note: WV = work value. Ref.: Integrated occupations. T-statistics in parentheses. Control variables: parental education, country. Asterisks refer to significance of coefficient (* p<0.05, ** p<0.01, *** p<0.001). Bold lettering indicates a significant difference compared to the reduced model (p<0.05). | | | |

| *Table S15:* Comparing Subgroup Coefficients Of The Reduced Multinomial Model Including Only Control Variables Versus Models That Additionally Include Gender Ideology, Educational Aspirations, And Work Values (Ref.: Non-Immigrant Girls) – Germany | | | |
| --- | --- | --- | --- |
|  | Masculine | Feminine | Ultra-feminine |
| **Non-immigrant boys** |  |  |  |
| Reduced model | 0.511*** | -0.196*** | -0.263*** |
|  | (16.92) | (-5.87) | (-10.54) |
| Incl. gender ideology | 0.513*** | -0.196*** | -0.268*** |
|  | (17.04) | (-5.66) | (-9.97) |
| Incl. educational aspirations | 0.500*** | -0.185*** | -0.273*** |
|  | (16.98) | (-5.54) | (-11.25) |
| Incl. WV: high income | 0.514*** | -0.199*** | -0.254*** |
|  | (17.12) | (-5.97) | (-10.29) |
| Incl. WV: helping others | 0.498*** | -0.196*** | **-0.236***** |
|  | (15.84) | (-5.78) | **(-9.71)** |
| Incl. WV: thinking/solving problems | 0.511*** | -0.196*** | -0.264*** |
|  | (16.89) | (-5.85) | (-10.54) |
| Incl. all mediators | 0.485*** | -0.184*** | -0.236*** |
|  | (15.38) | (-5.26) | (-9.16) |
| **Immigrant-origin boys** |  |  |  |
| Reduced model | 0.386*** | -0.150*** | -0.261*** |
|  | (8.35) | (-3.36) | (-10.10) |
| Incl. gender ideology | 0.388*** | -0.149*** | -0.265*** |
|  | (8.42) | (-3.34) | (-9.58) |
| Incl. educational aspirations | 0.392*** | -0.149*** | -0.268*** |
|  | (8.07) | (-3.53) | (-10.63) |
| Incl. WV: high income | **0.405***** | -0.155*** | **-0.249***** |
|  | **(8.73)** | (-3.46) | **(-9.59)** |
| Incl. WV: helping others | 0.381*** | -0.156*** | -0.239*** |
|  | (8.14) | (-3.46) | (-9.87) |
| Incl. WV: thinking/solving problems | 0.382*** | -0.146** | -0.261*** |
|  | (8.23) | (-3.29) | (-10.11) |
| Incl. all mediators | 0.394*** | -0.153*** | -0.234*** |
|  | (8.41) | (-3.51) | (-8.95) |
| **Immigrant-origin girls** |  |  |  |
| Reduced model | 0.028 | 0.030 | -0.065 |
|  | (0.87) | (0.65) | (-1.66) |
| Incl. gender ideology | 0.029 | 0.031 | -0.069 |
|  | (0.90) | (0.67) | (-1.75) |
| Incl. educational aspirations | 0.031 | 0.021 | -0.055 |
|  | (0.92) | (0.48) | (-1.45) |
| Incl. WV: high income | 0.030 | 0.025 | -0.049 |
|  | (0.93) | (0.54) | (-1.22) |
| Incl. WV: helping others | 0.035 | 0.030 | -0.086* |
|  | (1.01) | (0.65) | (-2.39) |
| Incl. WV: thinking/solving problems | 0.027 | 0.033 | -0.066 |
|  | (0.84) | (0.72) | (-1.69) |
| Incl. all mediators | 0.038 | 0.016 | -0.061 |
|  | (1.06) | (0.35) | (-1.71) |
| *N* | 2,329 | 2,329 | 2,329 |
| Note: WV = work value. Ref.: Integrated occupations. T-statistics in parentheses. Control variables: parental education, country. Asterisks refer to significance of coefficient (* p<0.05, ** p<0.01, *** p<0.001). Bold lettering indicates a significant difference compared to the reduced model (p<0.05). | | | |
|  | | | |

| *Table S16:* Comparing Subgroup Coefficients Of The Reduced Multinomial Model Including Only Control Variables Versus Models That Additionally Include Gender Ideology, Educational Aspirations, And Work Values (Ref.: Non-Immigrant Boys) – Netherlands | | | |
| --- | --- | --- | --- |
|  | Masculine | Feminine | Ultra-feminine |
| **Immigrant-origin boys** |  |  |  |
| Reduced model | -0.185 | -0.048 | 0.006 |
|  | (-1.42) | (-0.85) | (0.44) |
| Incl. gender ideology | -0.182 | -0.051 | 0.007 |
|  | (-1.40) | (-0.88) | (0.50) |
| Incl. educational aspirations | -0.144 | -0.053 | 0.007 |
|  | (-1.19) | (-0.96) | (0.49) |
| Incl. WV: high income | -0.157 | -0.038 | 0.007 |
|  | (-1.19) | (-0.62) | (0.47) |
| Incl. WV: helping others | -0.193 | -0.053 | 0.005 |
|  | (-1.49) | (-0.92) | (0.36) |
| Incl. WV: thinking/solving problems | -0.186 | -0.049 | 0.006 |
|  | (-1.43) | (-0.89) | (0.42) |
| Incl. all mediators | -0.142 | -0.056 | 0.009 |
|  | (-1.18) | (-0.98) | (0.58) |
| **Non-immigrant girls** |  |  |  |
| Reduced model | -0.462*** | 0.153*** | 0.356*** |
|  | (-9.42) | (3.46) | (9.49) |
| Incl. gender ideology | -0.461*** | 0.137** | 0.379*** |
|  | (-9.46) | (2.89) | (9.26) |
| Incl. educational aspirations | -0.441*** | 0.147*** | 0.365*** |
|  | (-9.41) | (3.39) | (9.76) |
| Incl. WV: high income | -0.468*** | 0.145** | 0.354*** |
|  | (-9.41) | (3.27) | (9.34) |
| Incl. WV: helping others | -0.471*** | **0.148**** | **0.324***** |
|  | (-9.71) | **(3.24)** | **(9.38)** |
| Incl. WV: thinking/solving problems | -0.464*** | 0.154*** | 0.361*** |
|  | (-9.47) | (3.50) | (9.62) |
| Incl. all mediators | -0.440*** | 0.132** | 0.385*** |
|  | (-9.28) | (2.82) | (9.45) |
| **Immigrant-origin girls** |  |  |  |
| Reduced model | -0.527*** | 0.383*** | 0.225*** |
|  | (-12.38) | (5.15) | (3.89) |
| Incl. gender ideology | -0.524*** | 0.370*** | 0.238*** |
|  | (-12.01) | (4.89) | (3.98) |
| Incl. educational aspirations | -0.503*** | 0.366*** | 0.259*** |
|  | (-11.98) | (5.06) | (4.07) |
| Incl. WV: high income | -0.531*** | 0.381*** | 0.226*** |
|  | (-12.41) | (5.27) | (3.82) |
| Incl. WV: helping others | **-0.537***** | **0.365***** | **0.181***** |
|  | **(-12.15)** | **(4.87)** | **(3.40)** |
| Incl. WV: thinking/solving problems | -0.526*** | 0.382*** | 0.208*** |
|  | (-12.19) | (5.22) | (3.91) |
| Incl. all mediators | -0.501*** | 0.358*** | 0.266*** |
|  | (-11.34) | (4.80) | (4.17) |
| *N* | 1,914 | 1,914 | 1,914 |
| Note: WV = work value. Ref.: Integrated occupations. T-statistics in parentheses. Control variables: parental education, country. Asterisks refer to significance of coefficient (* p<0.05, ** p<0.01, *** p<0.001). Bold lettering indicates a significant difference compared to the reduced model (p<0.05). | | | |

| *Table S17:* Comparing Subgroup Coefficients Of The Reduced Multinomial Model Including Only Control Variables Versus Models That Additionally Include Gender Ideology, Educational Aspirations, And Work Values (Ref.: Non-Immigrant Girls) – Netherlands | | | |
| --- | --- | --- | --- |
|  | Masculine | Feminine | Ultra-feminine |
| **Non-immigrant boys** |  |  |  |
| Reduced model | 0.462*** | -0.153*** | -0.356*** |
|  | (9.42) | (-3.46) | (-9.49) |
| Incl. gender ideology | 0.461*** | -0.137** | -0.379*** |
|  | (9.46) | (-2.89) | (-9.26) |
| Incl. educational aspirations | 0.441*** | -0.147*** | -0.365*** |
|  | (9.41) | (-3.39) | (-9.76) |
| Incl. WV: high income | 0.468*** | -0.145** | -0.354*** |
|  | (9.41) | (-3.27) | (-9.34) |
| Incl. WV: helping others | 0.471*** | **-0.148**** | **-0.324***** |
|  | (9.71) | **(-3.24)** | **(-9.38)** |
| Incl. WV: thinking/solving problems | 0.464*** | -0.154*** | -0.361*** |
|  | (9.47) | (-3.50) | (-9.62) |
| Incl. all mediators | 0.440*** | -0.132** | -0.385*** |
|  | (9.28) | (-2.82) | (-9.45) |
| **Immigrant-origin boys** |  |  |  |
| Reduced model | 0.277* | -0.202*** | -0.349*** |
|  | (2.16) | (-3.51) | (-8.77) |
| Incl. gender ideology | 0.279* | -0.188** | -0.371*** |
|  | (2.17) | (-3.22) | (-8.66) |
| Incl. educational aspirations | 0.297* | -0.201*** | -0.357*** |
|  | (2.50) | (-3.54) | (-8.90) |
| Incl. WV: high income | 0.312* | -0.183** | -0.346*** |
|  | (2.37) | (-2.92) | (-8.53) |
| Incl. WV: helping others | 0.278* | -0.202*** | -0.318*** |
|  | (2.20) | (-3.51) | (-8.49) |
| Incl. WV: thinking/solving problems | 0.278* | -0.203*** | -0.355*** |
|  | (2.16) | (-3.64) | (-8.97) |
| Incl. all mediators | 0.298* | -0.189** | -0.375*** |
|  | (2.51) | (-3.23) | (-8.73) |
| **Immigrant-origin girls** |  |  |  |
| Reduced model | -0.064 | 0.229** | -0.131 |
|  | (-1.90) | (3.07) | (-1.91) |
| Incl. gender ideology | -0.062 | 0.233** | -0.140* |
|  | (-1.90) | (3.12) | (-1.99) |
| Incl. educational aspirations | -0.062 | 0.219** | -0.106 |
|  | (-1.73) | (2.99) | (-1.45) |
| Incl. WV: high income | -0.063 | 0.236** | -0.128 |
|  | (-1.87) | (3.25) | (-1.82) |
| Incl. WV: helping others | -0.066 | 0.217** | -0.143* |
|  | (-1.92) | (2.97) | (-2.30) |
| Incl. WV: thinking/solving problems | -0.061 | 0.228** | -0.153* |
|  | (-1.80) | (3.11) | (-2.38) |
| Incl. all mediators | -0.060 | 0.225** | -0.119 |
|  | (-1.72) | (3.02) | (-1.60) |
| *N* | 1,914 | 1,914 | 1,914 |
| Note: WV = work value. Ref.: Integrated occupations. T-statistics in parentheses. Control variables: parental education, country. Asterisks refer to significance of coefficient (* p<0.05, ** p<0.01, *** p<0.001). Bold lettering indicates a significant difference compared to the reduced model (p<0.05). | | | |
|  | | | |

| *Table S18:* Comparing Subgroup Coefficients Of The Reduced Multinomial Model Including Only Control Variables Versus Models That Additionally Include Gender Ideology, Educational Aspirations, And Work Values (Ref.: Non-Immigrant Boys) – Sweden | | | |
| --- | --- | --- | --- |
|  | Masculine | Feminine | Ultra-feminine |
| **Immigrant-origin boys** |  |  |  |
| Reduced model | -0.082* | 0.096* | 0.008 |
|  | (-1.98) | (2.49) | (0.50) |
| Incl. gender ideology | -0.087* | 0.109** | 0.003 |
|  | (-2.10) | (2.70) | (0.27) |
| Incl. educational aspirations | **-0.041** | 0.076* | **0.006** |
|  | **(-1.02)** | (2.03) | **(0.39)** |
| Incl. WV: high income | -0.082* | 0.096* | 0.009 |
|  | (-1.97) | (2.46) | (0.54) |
| Incl. WV: helping others | -0.063 | 0.088* | 0.003 |
|  | (-1.53) | (2.29) | (0.19) |
| Incl. WV: thinking/solving problems | -0.084* | 0.097* | 0.009 |
|  | (-2.03) | (2.49) | (0.57) |
| Incl. all mediators | **-0.044** | 0.088* | 0.002 |
|  | **(-1.08)** | (2.26) | (0.16) |
| **Non-immigrant girls** |  |  |  |
| Reduced model | -0.448*** | 0.157*** | 0.185*** |
|  | (-17.86) | (5.99) | (9.41) |
| Incl. gender ideology | -0.446*** | 0.130*** | **0.208***** |
|  | (-17.23) | (4.79) | **(9.46)** |
| Incl. educational aspirations | **-0.412***** | 0.140*** | **0.185***** |
|  | **(-15.96)** | (5.20) | **(9.04)** |
| Incl. WV: high income | -0.447*** | 0.155*** | 0.178*** |
|  | (-17.68) | (5.84) | (9.11) |
| Incl. WV: helping others | -0.429*** | 0.151*** | 0.174*** |
|  | (-16.51) | (5.69) | (8.99) |
| Incl. WV: thinking/solving problems | -0.446*** | 0.156*** | 0.179*** |
|  | (-17.77) | (5.92) | (9.08) |
| Incl. all mediators | **-0.415***** | 0.116*** | 0.209*** |
|  | **(-15.65)** | (4.20) | (9.30) |
| **Immigrant-origin girls** |  |  |  |
| Reduced model | -0.485*** | 0.222*** | 0.168*** |
|  | (-18.35) | (6.18) | (6.79) |
| Incl. gender ideology | -0.483*** | 0.213*** | 0.173*** |
|  | (-18.17) | (5.93) | (6.98) |
| Incl. educational aspirations | **-0.445***** | 0.201*** | **0.176***** |
|  | **(-15.78)** | (5.49) | **(6.51)** |
| Incl. WV: high income | -0.484*** | 0.221*** | 0.169*** |
|  | (-18.29) | (6.10) | (6.59) |
| Incl. WV: helping others | **-0.462***** | 0.219*** | 0.149*** |
|  | **(-16.47)** | (5.85) | (5.93) |
| Incl. WV: thinking/solving problems | -0.486*** | 0.220*** | 0.178*** |
|  | (-18.50) | (6.04) | (6.75) |
| Incl. all mediators | **-0.445***** | 0.195*** | 0.179*** |
|  | **(-15.75)** | (5.35) | (6.87) |
| *N* | 2,314 | 2,314 | 2,314 |
| Note: WV = work value. Ref.: Integrated occupations. T-statistics in parentheses. Control variables: parental education, country. Asterisks refer to significance of coefficient (* p<0.05, ** p<0.01, *** p<0.001). Bold lettering indicates a significant difference compared to the reduced model (p<0.05). | | | |

| *Table S19:* Comparing Subgroup Coefficients Of The Reduced Multinomial Model Including Only Control Variables Versus Models That Additionally Include Gender Ideology, Educational Aspirations, And Work Values (Ref.: Non-Immigrant Girls) – Sweden | | | |
| --- | --- | --- | --- |
|  | Masculine | Feminine | Ultra-feminine |
| **Non-immigrant boys** |  |  |  |
| Reduced model | 0.448*** | -0.157*** | -0.185*** |
|  | (17.86) | (-5.99) | (-9.41) |
| Incl. gender ideology | 0.446*** | -0.130*** | **-0.208***** |
|  | (17.23) | (-4.79) | **(-9.46)** |
| Incl. educational aspirations | **0.412***** | -0.140*** | **-0.185***** |
|  | **(15.96)** | (-5.20) | **(-9.04)** |
| Incl. WV: high income | 0.447*** | -0.155*** | -0.178*** |
|  | (17.68) | (-5.84) | (-9.11) |
| Incl. WV: helping others | 0.429*** | -0.151*** | -0.174*** |
|  | (16.51) | (-5.69) | (-8.99) |
| Incl. WV: thinking/solving problems | 0.446*** | -0.156*** | -0.179*** |
|  | (17.77) | (-5.92) | (-9.08) |
| Incl. all mediators | **0.415***** | -0.116*** | -0.209*** |
|  | **(15.65)** | (-4.20) | (-9.30) |
| **Immigrant-origin boys** |  |  |  |
| Reduced model | 0.366*** | -0.060 | -0.177*** |
|  | (9.69) | (-1.51) | (-7.80) |
| Incl. gender ideology | 0.359*** | -0.020 | **-0.204***** |
|  | (9.06) | (-0.49) | **(-8.15)** |
| Incl. educational aspirations | 0.371*** | -0.062 | -0.179*** |
|  | (10.15) | (-1.62) | (-7.88) |
| Incl. WV: high income | 0.364*** | -0.058 | -0.169*** |
|  | (9.42) | (-1.42) | (-7.46) |
| Incl. WV: helping others | 0.365*** | -0.062 | -0.171*** |
|  | (9.73) | (-1.56) | (-7.84) |
| Incl. WV: thinking/solving problems | 0.362*** | -0.059 | -0.169*** |
|  | (9.49) | (-1.46) | (-7.44) |
| Incl. all mediators | 0.370*** | **-0.027** | -0.206*** |
|  | (9.66) | **(-0.66)** | (-8.23) |
| **Immigrant-origin girls** |  |  |  |
| Reduced model | -0.036 | 0.065 | -0.016 |
|  | (-1.84) | (1.74) | (-0.58) |
| Incl. gender ideology | -0.036 | 0.083* | -0.035 |
|  | (-1.79) | (2.22) | (-1.16) |
| Incl. educational aspirations | -0.033 | 0.060 | -0.009 |
|  | (-1.50) | (1.64) | (-0.31) |
| Incl. WV: high income | -0.037 | 0.065 | -0.008 |
|  | (-1.89) | (1.72) | (-0.30) |
| Incl. WV: helping others | -0.033 | 0.068 | -0.024 |
|  | (-1.57) | (1.77) | (-0.86) |
| Incl. WV: thinking/solving problems | -0.039* | 0.063 | **-0.001** |
|  | (-1.98) | (1.66) | **(-0.03)** |
| Incl. all mediators | -0.030 | 0.078* | -0.029 |
|  | (-1.38) | (2.11) | (-0.94) |
| *N* | 2,314 | 2,314 | 2,314 |
| Note: WV = work value. Ref.: Integrated occupations. T-statistics in parentheses. Control variables: parental education, country. Asterisks refer to significance of coefficient (* p<0.05, ** p<0.01, *** p<0.001). Bold lettering indicates a significant difference compared to the reduced model (p<0.05). | | | |
|  | | | |
